# Comparing Intramedullary Nails versus Dynamic Hip Screws in the Treatment of Intertrochanteric Hip Fractures on Post-operative Rehabilitation Outcomes – A Systematic Review and Meta-Analysis

**DOI:** 10.1177/21514593251350490

**Published:** 2025-06-17

**Authors:** Chantal Backman, Ashley Lam, Rosie Papp, Aurelie Tonjock Kolle, Franciely Daina Engel, Wenshan Li, Soha Shah, Colleen Webber, Peter Tanuseputro, Marie-Cecile Domecq, Steve Papp

**Affiliations:** 1 Faculty of Health Sciences, 6363 University of Ottawa, ON, Canada; 2 Institut du Savoir Montfort, Ottawa, ON, Canada; 3 574443 Ottawa Hospital Research Institute, Ottawa, ON, Canada; 4 Bruyère Research Institute, Ottawa, ON, Canada; 5 Department of Family Medicine and Primary Care, 71020 The University of Hong Kong, Hong Kong; 6 Health Sciences Library, 6363 University of Ottawa, ON, Canada; 7 Faculty of Medicine, 6363 University of Ottawa, ON, Canada

**Keywords:** dynamic implants, intramedullary nails, hip fracture, intertrochanteric hip fracture, functional outcomes, rehabilitation

## Abstract

**Objective:**

We conducted a systematic review and meta-analysis to compare post-operative rehabilitation outcomes between two common treatments in patients who have suffered an intertrochanteric hip fracture: intramedullary nails vs dynamic hip screws.

**Methods:**

We searched MEDLINE, EMBASE, Cochrane Central Register of Controlled Trials, and Web of Science up to August 10, 2022. The inclusion criteria were defined as Population: adults (>18 years old); Interventions/Comparators: intramedullary nails and dynamic hip screws; Outcomes: function, quality of life and survival; and Study type: randomized controlled trials and non-randomized studies. A meta-analysis was performed, and the fixed-effect model was selected to pool the data for homogeneous studies (I^2^ < 50%) and the random effect model was selected for heterogeneity I^2^>50%. The *P*-value of less than 0.05 was considered statistically significant. A narrative synthesis was conducted on the remaining outcomes with insufficient data (ie, missing means, or standard deviations).

**Results:**

108 studies were included in the review. 42 studies had outcomes that were included in the meta-analysis. There were modest differences after sensitivity analysis for the Parker mobility score mean difference (MD) = 0.70, 95% CI [0.12, 1.28], T = 3.11, df = 5, *P* = 0.03, and Harris hip score (MD = 0.94, 95% CI [0.34, 1.54], T = 3.54, df = 9, *P* = 0.006) favoring the nails. There were no statistically significant differences in the Functional Independence Measure (FIM) (MD = −2.50, 95% CI [−6.46, 1.45], Z = 1.24, *P* = 0.22), the Barthel Index (MD = 2.66, 95% CI [−7.60, 12.92], T = 1,12, df = 2, *P* = 0.38), the generic quality of life (MD = 0.04, 95% CI [−0.08, 0.17], Z = 0.70, *P* = 0.49), the health-related quality of life (MD = −0.14, 95% CI [−3.57, 3.28], Z = 0.08, *P* = 0.93) or mortality (1.00, 95% CI [0.96, 1.03], Z = 0.16, *P* = 0.87) outcomes.

**Conclusion:**

This review showed some differences in functional outcomes in the treatment of intertrochanteric fractures favoring intramedullary nails over dynamic hip screws. There were no differences between the groups for quality of life and mortality outcomes. The narrative synthesis showed additional outcomes that warrant further investigations.

## Background

Hip fractures are a significant medical concern worldwide,^1^ with an estimated global yearly incidence of 4.5 million by 2050.^2^ Intertrochanteric hip fractures typically result from trauma or falls and predominantly affect older adults because of decreased bone mineral density.^3^ Currently, the most common treatment options for intertrochanteric fractures include either intramedullary nails (IMN) or a dynamic hip screw (DHS) construct. The DHS is a plate and sliding screw construct used to stabilize the fracture, which can also be referred to as a sliding hip screw or a compression hip screw. These are considered ‘dynamic’ implants as they have the capacity for sliding at the plate/screw junction to allow for collapse at the fracture site.^4^ The DHS operates on the tension band principle, permitting the screw to move within the barrel, facilitating fracture compression as the patient starts to weight bear.^5^ Conversely, an IMN is a metal rod inserted into the medullary cavity of a bone, spanning across the fracture to provide a more medialized construct for support of the fracture.^6^ Biomechanically, the IMN is superior to the DHS.^7^ Several newer IMN, including gamma nails, intramedullary hip screw, and proximal femoral nails, also facilitate dynamic movement and thus collapsing at the fracture site.^8^ The DHS was considered the gold standard treatment for intertrochanteric fractures in the past,^4,9^ but the IMN has been a preferable form of treatment more recently.^10^

Although there have been meta-analyses and systematic reviews comparing the two methods to determine the ideal implant, there have been inconsistencies in their findings. These variations may come from the diverse range of outcomes reported as studies look at parameters within perioperative outcomes and postoperative complications. Some studies have favored IMN implants when considering perioperative outcomes^11–14^ such as operative blood loss and operative time. Parker and Pryor’s meta-analysis showed that while many of the outcomes analyzed^15–19^ showed no significant difference, recipients of DHS had a reduced risk for femoral shaft fractures or reoperation rate than IMN.^20^ However, no previous reviews have specifically focused on longer term (ie, 12-month post-surgery) functional outcomes, quality of life, and mortality rates for these treatment options. The overall goal was to systematically compare post-operative rehabilitation outcomes (including functional, quality of life, and survival) between two groups of IMN vs DHS fixations in the treatment of intertrochanteric fractures.

## Methods

### Study Design

We conducted a systematic review and meta-analysis according to the Preferred Reporting Items for Systematic Reviews and Meta-Analysis (PRISMA) guidelines^21^ and Cochrane’s Handbook for Systematic Reviews of Interventions.^22^ The protocol was developed, registered in PROSPERO (CRD# CRD42022364556) and published.^23^

### Protocol Amendments

The review was conducted as planned, with no deviations from the registered protocol. However, due to the considerable number of outcomes found, we focused this systematic review on post-operative functional, quality of life, and survival outcomes.

### Eligibility Criteria

#### Population

This review considered studies in adult patients (18+) with an intertrochanteric hip fracture (AO/OTA classification: type 31A).^3,24–27^

#### Interventions/Comparators

Studies comparing the effectiveness of IMN and DHS were included. IMN includes double lag screw nails, Targon nail, proximal femoral nail, long or short cephamedullary nail, gamma nail, InterTan, Profin nail, Kuntscher-Y nail, intramedullary hip screw, holland nail, and ace nail. DHS includes sliding hip screws, vari-angle hip screw, and compression hip screw. We excluded studies that evaluated uncommon methods of fixation such as ender nail, nystrom nail, Grosse-Kempf nail, Moore’s pins, transverse proximal screw, dynamic condylar screw, contralateral reverse distal femoral locking compression plate, and less invasive stabilization system. Studies that had no comparator group or that compared two nails, or two screws were also excluded.

#### Outcome Measures

The studies included in the review identified at least one of the following outcomes at 12-month post-surgery: functional outcomes (ie, Harris hip score, Parker mobility score, Functional Independence Measure (FIM), etc.), quality of life, and survival outcomes.

#### Types of Studies

We included randomized controlled trials (RCTs) and non-randomized studies (eg, cohort, case-control, controlled before-and-after, interrupted-time-series, and controlled trials not using full randomization).

### Search Strategy

A systematic search strategy was conducted in MEDLINE (Ovid), EMBASE (Ovid), Cochrane Central Register of Controlled Trials (Ovid), and Web of Science from their date of inception to August 10, 2022. The search strategy was developed by an experienced health sciences librarian (MCD) and subsequently peer-reviewed by a second information specialist using the Peer Review or Electronic Search Strategies guideline.^28^ The search parameters, which included terms like hip fractures, intertrochanteric fracture, dynamic, screw, bone, fixation, intramedullary, and others, were adjusted to target articles relevant to orthopedic interventions and their associated outcomes. The search strategies are described in the supplemental file #1. References were exported into Covidence (https://www.covidence.org) and duplicates were removed.

### Screening and Data Extraction

Three reviewers (AL, RP and ATK) independently screened the references in a two-step approach (title and abstract, then full text) according to the eligibility criteria. Throughout the screening process, CB and SP were available to address any reviewer disagreements. This collaborative approach ensured a comprehensive evaluation of the eligibility criteria. Three reviewers (AL, RP and ATK) independently extracted and organized the data using a previously piloted data extraction sheet. The following were extracted for each included study: author, country, study design, intervention 1, intervention 2, patients (n) in each group, outcomes/results, follow-up period.

### Risk of Bias Assessment

During the data extraction process, the risk of bias of the included studies was independently assessed by three authors (AL RP, ATK). The Risk of Bias 2 (RoB 2) and the Risk of Bias in Non-randomized Studies - of Interventions (ROBINS-I) were used in the assessment. RoB 2 is an updated version of the Cochrane risk-of-bias tool specifically designed for RCTs.^29^ It evaluates domains such as randomization, deviations from interventions, missing outcome data, measurement of outcomes, and selection of reported results.^29^ Questions are employed within each domain to determine the level of bias, classifying it as low risk, some concerns, or high risk. ROBINS-I, however, is intended for non-randomized studies comparing interventions.^30^ It assesses domains such as confounding, selection bias, and measurement of interventions and outcomes.^30^ Similar to RoB 2, ROBINS-I utilizes signalling questions to assign a judgment of bias within each domain such as critical risk, serious risk, moderate risk, or low risk. At the end of the quality assessment, we used Robvis, a web-based application specifically designed for visualizing risk-of-bias assessments in systematic reviews to report the findings.^31^

### Data Analysis

The Cochrane Review Manager 7.9.2 (RevMan Web) was used to conduct the meta-analysis.^32^ For the analysis of continuous variables, we utilized the inverse variance method to calculate the mean difference (MD) along with its corresponding 95% confidence interval (CI). For the analysis of dichotomous outcomes, we employed the Mantel-Haenszel method to calculate the pooled odds ratio (OR) as well as its corresponding 95% confidence interval (CI). Heterogeneity was assessed with the I^2^. If, for a given outcome, there was significant heterogeneity (I^2^ > 50%) and the number of included studies in the analysis was > 5, we selected a random effects model to pool the data. On the contrary, if I^2^ < 50% or the number of included studies in the analysis was <5, the fixed-effect model was selected.^33^ Sensitivity analysis was performed to explore the source of heterogeneity.^34^ Publication bias was investigated by funnel plot and an asymmetric plot suggested possible publication bias.^34^ All *P*-values were two-sided and a *P*-value of less than 0.05 was considered statistically significant. Findings from studies with insufficient data (ie, missing means, or standard deviations) were excluded from the meta-analysis, but were grouped by outcome and synthesized narratively.

## Results

### Search Results

**Figure 1.**
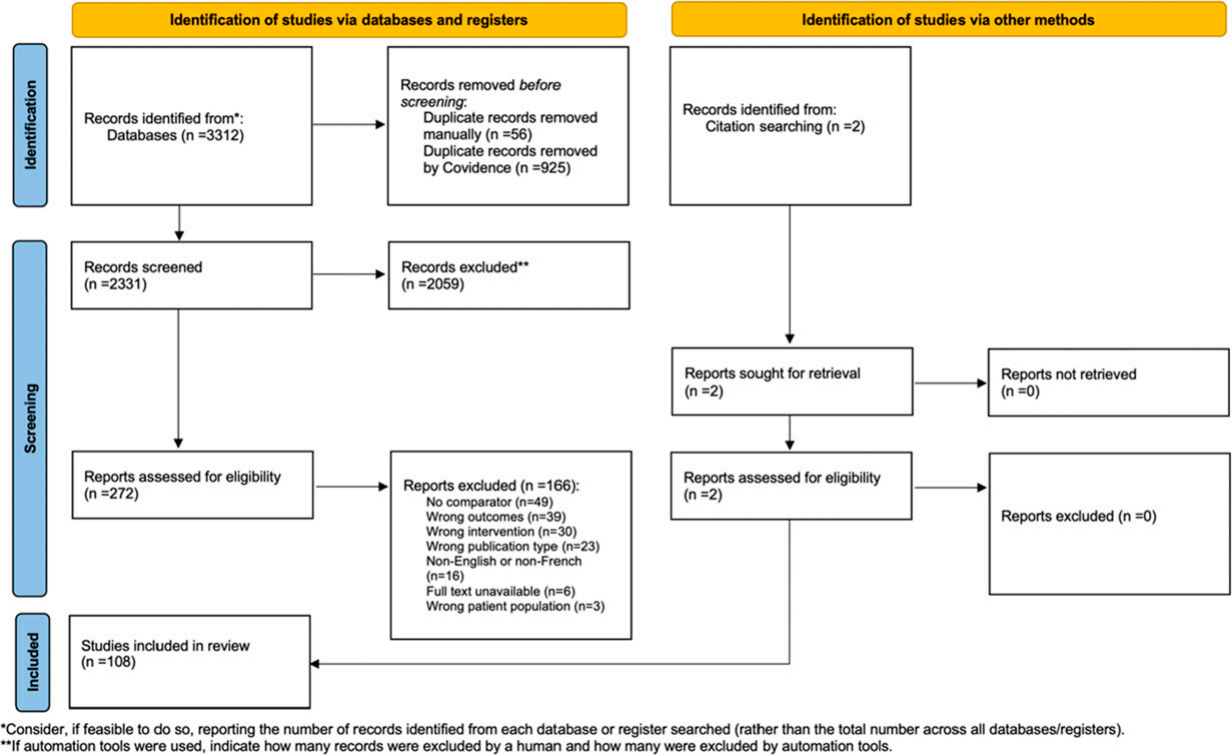
PRISMA Flowchart

From the initial database searches, 3312 studies were identified. After duplicate records were removed by Covidence (n = 925) or manually (n = 56), the titles and abstracts were screened of the remaining studies (n = 2331) based on the inclusion criteria. A full-text review of 272 studies was completed, which identified 166 studies that did not meet the inclusion criteria and were therefore excluded. Reasons for exclusion included no comparator (n = 49), wrong outcomes (n = 39), wrong intervention (n = 30), wrong publication type (n = 23), non-English or non-French (n = 16), full text unavailable (n = 6), and wrong patient population (n = 3). All studies that did not meet the criteria are listed in Supplemental file 2 with the reason for exclusion. The remaining 106 met our inclusion criteria. Two additional studies were identified through manual citation searching, which met the inclusion criteria. Thus, 108 studies were included in the review (Figure 1).

### Study Characteristics

This 108 included studies were conducted between 1988 and 2023. Studies took place in a range of countries, including the highest frequency of studies in India (n = 21), China (n = 16), and United Kingdom (n = 13). Most study designs were RCTs (n = 52, 48.1%), prospective cohort studies (n = 25, 23.1%) and retrospective cohort studies (n = 24, 22.2%). See Table 1 for further details on the characteristics of studies.

**Table 1.** Study Characteristics (n = 108)

Author	Country	Study objectives	Study Design	Intervention 1: Nails	Intervention 2: Screws
Adams 2001^35^	United Kingdom	To compare the surgical complications and functional outcome of the Gamma nail intramedullary fixation device versus the Richards sliding hip screw and plate device in intertrochanteric femoral fractures	RCT	Gamma nail	Richards sliding hip screw and plate device
Adeel 2020^a^ ^36^	Pakistan	To compare DHS with PFN for the treatment of AO types A2 and A3 per-trochanteric fractures of femur	RCT	Proximal femoral nail (PFN)	Dynamic hip screw (DHS)
Adulkasem 2021^37^	Thailand	To examine the roles of both clinical and surgical parameters as prognostic factors on 1-year postoperative ambulatory outcomes, reaching a good functional outcome (the New Mobility Score: NMS >= 5) and returning to preinjury functional status at one year, of older patients with intertrochanteric fracture	Retrospective cohort study	Proximal femoral nail antirotation (PFNA)	Dynamic hip screw (DHS)
Ahmad 2019^38^	Pakistan	To compare clinical, functional and radiological outcome scores of patients sustaining neck of femur (NOF) and intertrochanteric (IT) fractures at defined follow-up visits treated with different surgical procedures	Prospective cohort study	Intramedullary (IM) nail	Dynamic hip screw (DHS)
Ahrengart 2002^39^	Sweden	To compare the compression hip screw with the Gamma nail in the treatment of intertrochanteric fractures	RCT	Gamma nail	Compression hip screw (CHS)
Aktselis 2014^a^ ^40^	Greece	To assess whether an intramedullary nail is superior to a sliding hip screw in the treatment of multifragmentary intertrochanteric fractures	RCT	Gamma nail	Sliding hip screw (AMBI)
Alessio-Mazzola 2022^a^ ^41^	Italy	To compare perioperative results of sliding hip screws and intramedullary nails for two-part femoral fractures through the analysis of transfusion rates, postoperative blood loss, midterm outcomes, and direct costs	Prospective cohort study	Proximal Femoral Nail (PFN)	Sliding hip screw (SHS)
Amini 2017^42^	Not reported	To examine treatment outcomes and complications in patients who sustained high-energy intertrochanteric fractures treated with SHSs and CMNs	Retrospective cohort study	Cephalomedullary nails	Sliding hip screw (SHS)
Andalib 2020^43^	Iran	To compare the efficacy of EM and IM fixation in treatment of unstable intertrochanteric fractures	RCT	Cephalomedullary nails	Dynamic hip screw (DHS)
Anjum 2020^44^	Pakistan	To evaluate and compare the post-operative functional outcome of patients of intertrochanteric fractures of femur undergoing either dynamic hip screw (DHS) or proximal femoral nail (PFN) fixation presenting under spinal anesthesia in tertiary care hospital	RCT	Proximal femoral nail (PFN)	Dynamic hip screw (DHS)
Anshul 2022^45^	India	to evaluate the functional and radiological outcome of DHS and PFN in treatment of intertrochanteric fractures with special reference to surgical site infection.	Prospective cohort study	Proximal femoral nail (PFN)	Dynamic hip screw (DHS)
Aros 2008^a^ ^46^	United States	To determine whether patients who sustain an intertrochanteric fracture have better outcomes when stabilized using a sliding hip screw or an intramedullary nail	Prospective cohort study	Intramedullary (IM) nail	Sliding hip screw (SHS)
Bajpai 2019^a^ ^47^	India	To compare the functional outcome of patients of unstable proximal femoral fractures treated using either DHS or proximal femoral nail	RCT	Proximal femoral nail (PFN)	Dynamic hip screw (DHS)
Balusamy 2017^48^	India	To compare the functional and radiological outcome of pertrochanteric fractures in elderly patients treated with dynamic hip screw and proximal femoral nail	Prospective cohort study	Proximal femoral nail (PFN)	Dynamic hip screw (DHS)
Barton 2010^a^ ^49^	United Kingdom	To compare the outcome of treatment of these unstable fractures of the proximal part of the femur with either a sliding hip screw or a long gamma nail	RCT	Long gamma nail	Sliding hip screw (SHS)
Biakto 2022^50^	Indonesia	To compare postoperative walking ability in femoral intertrochanter fractures treated with DHS and PFNA and evaluated the confounding factors	Retrospective cross-sectional study	Proximal femoral nail antirotation (PFNA)	Dynamic hip screw (DHS)
Bienkowski 2006^51^	Canada	To compare the intramedullary nail device with the traditional dynamic hip screw	Prospective cohort study	Trochanteric Fixation Nail (TFN)	Dynamic hip screw (DHS)
Bridle 1991^52^	United Kingdom	To compare the fixation of intertrochanteric fractures of the proximal femur in elderly patients with random use of either a Dynamic Hip Screw (DHS) or a new intramedullary device, the Gamma nail	RCT	Gamma nail	Dynamic hip screw (DHS)
Butt 1995^53^	United Kingdom	To compare the results of treatment with a dynamic hip screw (DHS) and a gamma nail in patients with peri-trochanteric fractures of the femur	RCT	Gamma nail	Dynamic hip screw (DHS)
Cai 2016^54^	China	To explore the best way to treat stable intertrochanteric fractures, taking hidden blood loss into account	RCT	Intramedullary fixation	Extramedullary fixation
Canbeyli 2021^a^ ^55^	Turkey	To evaluate the possible effects of surgical procedures on mortality and to identify the possible risk factors for mortality in the management of geriatric hip fractures	Retrospective cohort study	Proximal femoral nail antirotation (PFNA)	Sliding hip screw (SHS)
Carulli 2017^a^ ^56^	Italy	To evaluate the clinical and radiological results of fixation of stable or minimal unstable trochanteric fractures with PFNa in a population of patients compared to a control group treated by SHS	Prospective cohort study	Proximal femoral nail antirotation (PFNA)	Sliding hip screw (SHS)
Chander 2015^57^	India	To compare the functional outcome of unstable intertrochanteric fractures treated with PFN and DHS	Prospective cohort study	Proximal femoral nail (PFN)	Dynamic hip screw (DHS)
Chechik 2014^a^ ^58^	Israel	To compare the functional and radiographic results of dynamic hip screw (DHS) and expandable proximal femoral nail (EPFN) in the treatment of extracapsular hip fractures	RCT	Expandable proximal femoral nail (EPFN)	Dynamic hip screw (DHS)
Chen 2018^59^	China	To compare the clinical efficacy of proximal femoral nail anti-rotation (PFNA) and dynamic hip screw (DHS) internal fixation for hip fracture in the elderly patients	RCT	Proximal femoral nail antirotation (PFNA)	Dynamic hip srew (dhs)
Cho 2016^a^ ^60^	Korea	To evaluate and compare the clinical and functional outcomes of dynamic hip screw (DHS) and proximal femoral nail antirotation (PFNA) treatment of AO type 1 intertrochanteric fractures in elderly patients	Retrospective cohort study	Proximal femoral nail antirotation (PFNA)	Dynamic hip screw (DHS)
Chua 2013^61^	Singapore	To compare the short-term ambulatory function of elderly patients after fixation of unstable intertrochanteric fractures with either the AO-ASIF proximal femoral nail anti-rotation (PFNA) device or the dynamic hip screw (DHS)	Retrospective cohort study	Proximal femoral nail antirotation (PFNA)	Dynamic hip screw (DHS)
Cordero-Ampuero 2021^a^ ^62^	Spain	To evaluate the influence of surgical quality (as evaluated in the post-surgical radiographic control) on mortality, complications and recovery of walking ability in patients older than 64 years with hip fracture	Retrospective cohort study	Trochanteric nails	Sliding hip screw (SHS)
Davis 1988^a^ ^63^	United Kingdom	To compare the use of the Kuntscher-Y nail and a sliding hip screw in the treatment of intertrochanteric fractures of the femur	RCT	Kuntscher-Y nail (K-Y)	Sliding hip screw (SHS)
De Groof 1988^64^	Belgium	To compare the dynamic hip screw implant system with the Bohler Nail-Plate Fixation	Retrospective cohort study	Böhler nail	Dynamic hip screw (DHS)
Duan 2017^65^	China	To compare the effects of proximal femoral nail antirotation (PFNA) and dynamic hip screw (DHS) in the treatment of intertrochanteric fractures in order to investigate the significance of internal fixation in the treatment of intertrochanteric fractures in the elderly	Prospective cohort study	Proximal femoral nail antirotation (PFNA)	Dynamic hip srew (DHS)
Dujardin 2001^66^	France	To compare the results obtained with a sliding screw plate and an experimental device including a small-diameter nail that can be placed with a mini-invasive approach and provides a stable fixation	RCT	Nail	Trochanteric hip screw (THS)
Dunne 2021^a^ ^67^	England	To investigate functional outcomes in patients who have undergone either sliding hip screw fixation or intramedullary fixation using varied lengths of nails to assess potential superiority	Retrospective cohort study	Intramedullary (IM) Nail	Sliding hip screw (SHS)
Duymus 2019^a^ ^68^	Turkey	To compare the clinical and radiological results of the surgical treatments of proximal femoral nail (PFN), dynamic hip screw (DHS) or proximal femoral locking compression plate (PF-LCP) in patients with AO 31A2.2/2.3 unstable intertrochanteric femoral fracture (ITF)	Retrospective cohort study	Proximal Femoral Nail (PFN)	Dynamic hip screw (DHS)
Elis 2012^a^ ^69^	Israel	To retrospectively assess our results of treating reverse oblique fracture with an expendable proximal femoral nail (EPFN) or with a dynamic condylar screw-plate (DCS: 95°)	Retrospective cohort study	Expendable proximal femoral nail (EPFN)	Dynamic condylar screw
Foss 2009^70^	Denmark	To evaluate the role of surgical technique on postoperative pain	Prospective cohort study	Intramedullary hip screws (IMHS)	Dynamic hip screw (DHS)
Foulongne 2009^71^	France	To assess the presumed advantages of this innovating and original system by comparing it with a DHS type (HowmedicaTM, Rutherford, NJ, USA) device in the management of trochanteric fractures in the elderly	Case-control study	Mini-invasive nailing device (BCM nail)	Dynamic hip screw (DHS)
Fritz 1999^72^	Germany	To compare the new intramedullary implant of the gliding nail to the gamma nail in the fixation of unstable trochanteric fractures in elderly patients	RCT	Gamma nail	Dynamic hip screw (DHS)
Fu 2020^73^	Taiwan	To compare the clinical outcomes of patients with the unstable type of intertrochanteric fractures treated with DHS+TSP or IMNing (proximal femoral nail antirotation (PFNA))	Retrospective cohort study	Proximal femoral nail antirotation (PFNA)	Dynamic hip screw + Trochanteric Stabilizing Plate (DHS + TSP)
Garg 2022^a^ ^74^	India	To compare the role of the various surgical modalities ie, Hemiarthroplasty (HA), Dynamic Hip Screw (DHS), Cephalo-medullary nail (CMN) in the management of intertrochanteric fractures in elder patients with comparison of the results and assessment of the complications encountered with each method	Prospective cohort study	Proximal femoral nail (PFN)	Dynamic hip screw (DHS)
Giraud 2005^75^	France	To compare the dynamic hip screw (Synthes) and intramedullary fixation (Targon PF, Aesculap) for the treatment of pertrochanteric fractures in terms of stability, complications and cost effectiveness	RCT	Targon nail	Dynamic hip srew (DHS)
Grønhaug 2022^a^ ^76^	Norway	To investigate if there are differences in outcome between sliding hip screws (SHSs) and intramedullary nails (IMNs) with regard to fracture stability	Prospective cohort study	Intramedullary (IM) nail	Sliding hip screw (SHS)
Guerra 2014^a^ ^77^	Brazil	To compare the functional recovery at 1-year follow-up of elderly patients with intertrochanteric fractures treated surgically with the dynamic hip screw (DHS) or proximal femoral nail (PFN) fixation techniques	RCT	Proximal Femoral Nail (PFN)	Dynamic hip screw (DHS)
Guo 2013^78^	China	To compare the clinical effectiveness of the percutaneous compression plate (PCCP) versus proximal femoral nail anti-rotation (PFNA) in the treatment of intertrochanteric fractures in elderly patients	RCT	Proximal femoral nail antirotation (PFNA)	Percutaneous compression plate (PCCP)
Guo 2017^79^	China	To compare efficacy and long-term prognosis with proximal femoral nail anti-rotation (PFNA) and dynamic hip screw (DHS) for the treatment of unstable intertrochanteric fractures	Retrospective cohort study	Proximal femoral nail antirotation (PFNA)	Dynamic hip srew (DHS)
Han 2021^80^	China	To explore the clinical efficacy and safety of proximal femoral nail antirotation (PFNA) and dynamic hip screw (DHS) in the treatment of unstable intertrochanteric fractures in elderly patients	Cohort study	Proximal femoral nail antirotation (PFNA)	Dynamic hip srew (DHS)
Hardy 1998^a^ ^81^	Belgium	To compare the use of an intramedullary hip-screw with the use of a compression hip screw with a plate in elderly patients who had an intertrochanteric fracture	RCT	Intramedullary hip screw (IMHS)	Compression hip screw with a plate
Harrington 2002^82^	United Kingdom	To compare a standard sliding hip screw and the intramedullary hip screw for the treatment of unstable intertrochanteric fractures in the elderly	RCT	Intramedullary hip screw (IMHS)	Compression hip screw
Henzman 2015^a^ ^83^	United States	To evaluate the incidence of and complications associated with the use of an intramedullary nail vs open reduction and internal fixation (ORIF) with a sliding compression hip screw and plate in treating intertrochanteric fractures	Retrospective cohort study	Intramedullary (IM) nail	Sliding hip screw (SHS)
Hoffman 1996^84^	Australia	To compare the Ambi hip screw and the Gamma nail for the treatment of 69 patients over the age of 50 years with intertrochanteric femoral fractures	RCT	Gamma nail	Ambi hip screw
Huang 2015^85^	China	To compare the clinical effectiveness of proximal femoral locking compression plate (PFLCP), dynamic hip screw (DHS), and proximal femoral nail antirotation (PFNA) for unstable intertrochanteric femoral fracture treatment	RCT	Proximal femoral nail antirotation (PFNA)	Dynamic hip srew (DHS)
Jain 2015^a^ ^86^	India	To compare the effectiveness of dynamic hip screw (DHS) with a proximal femoral nail (PFN) in patients admitted with intertrochanteric fractures	RCT	Proximal femoral nail (PFN)	Dynamic hip srew (DHS)
Jia 2017^a^ ^87^	China	To compare the effect of dynamic hip screw (DHS) and proximal femoral nail anti-rotation (PFNA) in the treatment of intertrochanteric femoral fractures of elderly subjects and evaluated the effect of PFNA internal fixation	Retrospective cohort study	Proximal femoral nail antirotation (PFNA)	Dynamic hip srew (DHS)
Jonnes 2016^88^	India	To compare the functional and radiological outcome of PFN with DHS in treatment of Type II intertrochanteric fractures	Prospective cohort study	Proximal femoral nail (PFN)	Dynamic hip screw (DHS)
Khan 2021^a^ ^89^	Pakistan	To compare the dynamic hip screw with proximal femoral nail technique in intertrochanteric femur fracture patients	Retrospective cohort study	Proximal femoral nail (PFN)	Dynamic hip screw (DHS)
Koduru 2016^90^	India	To compare the clinical and radiological outcomes of Proximal femoral nail and Dynamic hip screw fixation	Prospective cohort study	Proximal femoral nail (PFN)	Dynamic hip screw (DHS)
Kumar 2012^a^ ^91^	India	To compare the outcome of intertrochanteric fractures treated with Dynamic Hip Screw and Proximal Femoral nail	RCT	Proximal femoral nail (PFN)	Dynamic hip srew (DHS)
Kyavater 2015^92^	India	To compare the clinical and radio graphical results of the DHS and PFN for the treatment of intertrochanteric hip fractures (Load bearing vs Load shearing)	Prospective cohort study	Proximal femoral nail (PFN)	Dynamic hip screw (DHS)
Leung 1992^93^	United Kingdom	To report a randomised prospective study of i86 fractures treated by either the Gamma nail or a dynamic hip screw.	RCT	Gamma nail	Dynamic hip srew (DHS)
Li 2021^94^	China	To compare the effects of proximal femoral nail anti-rotation (PFNA) and dynamic hip screw (DHS) helical blade treatments in patients with osteoporotic femoral intertrochanteric fractures	RCT	Proximal femoral nail antirotation (PFNA)	Dynamic hip srew (DHS)
Little 2008^a^ ^95^	United Kingdom	To compare the outcome of patients treated for an intertrochanteric fracture of the femoral neck with a locked, long intramedullary nail with those treated with a dynamic hip screw (DHS)	RCT	Holland nail	Dynamic hip screw (DHS)
Matre 2013a^96^	Norway	To clarify the distinctions between these two implants, we studied a large group of patients with simple two-part fractures and specifically asked whether patients with simple two-part intertrochanteric fractures treated with IMNing had (1) lower risks of reoperation and (2) less pain and better quality of life than patients treated with SHSs	Prospective cohort study	Intramedullary (IM) nail	Sliding hip screw (SHS)
Matre 2013b^97^	Norway	To compare IMNs and SHS in the treatment of transverse/reverse oblique trochanteric and subtrochanteric fractures using data from the Norwegian Hip Fracture Register (NHFR)	Retrospective cohort study	Intramedullary (IM) nail	Sliding hip screw (SHS)
Matre 2013c^a^ ^98^	Norway	To assess whether use of the TRIGEN INTERTAN nail, as compared with a sliding hip screw, resulted in less postoperative pain, improved functional mobility, and reduced surgical complication rates for patients with an intertrochanteric or subtrochanteric fracture	RCT	Trochanteric antegrade nail (InterTan)	Sliding hip screw (SHS)
McLaren 1991^99^	United Kingdom	To investigate whether the above theoretical advantages and disadvantages of the Pugh nail-plate when compared with the DHS were significant in practice	RCT	Pugh nail	Dynamic hip screw (DHS)
Mohan 2019^100^	India	To compare the operative parameters and functional outcome of two-part intertrochanteric fractures treated by dynamic hip screw (DHS) and proximal femur nail (PFN)	Retrospective cohort study	Proximal femoral nail (PFN)	Dynamic hip screw (DHS)
O'Brien 1995^101^	Canada	To compare the efficacy of the gamma nail (GN) to the dynamic hip screw (DHS) in the management of intertrochanteric hip fractures	RCT	Gamma nail	Dynamic hip srew (DHS)
Ong 2019^102^	United Kingdom	To determine if different patient groups have superior mobility regain following intertrochanteric hip fracture fixation with a cephomedullary nail compared to a sliding hip screw (SHS)	RCT	Cephalomedullary nail	Sliding hip screw (SHS)
Pajarinen 2005^103^	Finland	To assess the patients’ recovery after operative treatment of a pertrochanteric femoral fracture with either DHS or PFN, in a randomised, prospective series of 108 patients	RCT	Proximal femoral nail (PFN)	Dynamic hip screw (DHS)
Parker 2012^a^ ^104^	United Kingdom	To compare the Targon Proximal Femoral (PF) Nail (B. Brawn Ltd, Tuttlingen, Germany) with the SHS (Corin PLC, Cirencester, United Kingdom) in order to determine which method of surgical fixation is superior	RCT	Targon PF nail	Sliding hip screw (SHS)
Parker 2017^a^ ^105^	United Kingdom	To determine the optimum choice of implant for a patient with a different types of trochanteric hip fracture	RCT	Intramedullary (IM) nail	Sliding hip screw (SHS)
Patel 2021^106^	India	To compare functional and clinical outcome of dynamic hip screw (DHS) and intramedullary proximal femoral nail (PFN) in intertrochanteric femur fractures	Prospective cohort study	Proximal femoral nail (PFN)	Dynamic hip screw (DHS)
Pehlivanoglu 2021^107^	Turkey	To compare the cost -effectivity, clinical and radiological outcomes of the proximal femoral nail (PFN) and Dynamic hip screw (DHS) in the management of stable intertrochanteric femur fractures (SIFFs)	Retrospective cohort study	Proximal femoral nail (PFN)	Dynamic hip srew (DHS)
Prakash 2022^108^	India	To compare the functional outcome of the DHS and PFN for the treatment of intertrochanteric hip fractures achieved by the patient based on Harris hip score	RCT	Proximal femoral nail (PFN)	Dynamic hip srew (DHS)
Pundkar 2016^a^ ^109^	India	To compare the short-term result in DHS and PFN in all type of IT fracture and to set guidelines for managements of intertrochantric fractures	RCT	Proximal femoral nail (PFN)	Dynamic hip srew (DHS)
Radcliff 2012^a^ ^110^	United States	To examine trends in the use and associated outcomes of intramedullary nailing compared with sliding hip screws in Veterans Affairs (VA) hospitals	Retrospective cohort study	Intramedullary (IM) nail	Sliding hip screw (SHS)
Rao 2015^111^	India	To compare the outcome of intertrochanteric fractures treated with Dynamic Hip Screw and Proximal Femoral nail	Prospective cohort study	Proximal femoral nail (PFN)	Dynamic hip srew (DHS)
Reddy 2016^112^	India	To verify the theoretical advantages of the Recon nail over the dynamic hip screw device and also whether it actually alters the eventual functional outcome of the patient	Prospective cohort study	Reconstruction nail	Dynamic hip srew (DHS)
Reindl 2015^a^ ^113^	Canada	To compare the clinical and radiographic outcomes of patients who had been treated with a traditional extramedullary hip screw for an unstable (AO/OTA 31-A2) intertrochanteric hip fracture with those of patients who had been treated with the newer intramedullary device for the same injury	RCT	Intramedullary (IM) nail	Dynamic hip screw (DHS)
Rogmark 2010^114^	Sweden	To describe the changes in surgical treatment of femoral neck fractures and trochanteric hip fractures in Sweden over the period 1998-2007 and the regional differences in treatment	Retrospective cohort study	Intramedullary (IM) nail	Sliding hip screw (SHS)
Sanders 2017^a^ ^115^	Canada	To compare outcomes in elderly patients with intertrochanteric hip fractures treated with either the sliding hip screw (SHS) or InterTAN intramedullary device (IT)	RCT	InterTAN intramedullary device (IT)	Sliding hip screw (SHS)
Saudan 2002^a^ ^116^	Switzerland	To compare the results between a sliding compression hip screw and an intramedullary nail in the treatment of pertrochanteric fractures	RCT	Proximal femoral nail (PFN)	Dynamic hip screw (DHS)
Schemitsch 2023^a^ ^117^	Canada	To compare 1-year outcomes of patients with trochanteric fractures treated with the IMN vs an SHS	RCT	Intramedullary NAIL (gamma 3 nail)	Sliding hip screw (SHS)
Sevinc 2020 ^118^	Turkey	To compare clinical and functional outcomes between patients treated with Dynamic hip screw (DHS) and Proximal Femoral Nail-Antirotation (PFN-A) implants	Prospective cohort study	Proximal femoral nail antirotation (PFNA)	Dynamic HIP SCREW (DHS)
Sharma 2018^119^	India	To evaluate and compare the clinical and radiological outcomes of patients with stable intertrochanteric fractures treated with proximal femoral nail vs. dynamic hip screw	RCT	Proximal femoral nail (PFN)	Dynamic hip screw (DHS)
Shishodia 2017^120^	India	To compare the outcome of Proximal Femoral Nail and Dynamic Hip Screw in the treatment of intertrochanteric fractures	Prospective cohort study	Proximal femoral nail (PFN)	Dynamic hip srew (DHS)
Shivanna 2015^121^	India	To compare the surgical treatment of intertrochanteric fractures of the femur with the intramedullary device (Proximal femoral nail) with Dynamic Hip Screw device, with respect to: Union rates, mobilization/ ambulatory status, subjective parameters, objective parameters, radiological findings and complications	RCT	Proximal femoral nail (PFN)	Dynamic hip srew (DHS)
Singh 2021^122^	India	To assess the functional outcome of intertrochanteric fracture of femur treated with dynamic hip screw (DHS) with de-rotation screw comparing and proximal femoral nail (PFN)	RCT	Proximal femoral nail (PFN)	Dynamic hip screw with de-rotation screw (DHS with DRS)
Singh 2019^a^ ^123^	India	To compare the functional and radiological outcome of Proximal Femoral Nail anti-rotation-Asia (PFNA-II) and Dynamic Hip screw (DHS) used in fixation of stable (AO type 31 A1-A2.1) intertrochanteric fractures in elderly	RCT	Proximal femoral nail antirotation (PFNA-II)	Dynamic hip screw (DHS)
Suh 2015^a^ ^124^	Korea	To determine whether there were differences in clinical and radiologic outcomes among bipolar hemiarthroplasty (BH), compression hip screw (CHS) and proximal femur nail antirotation (PFNA) in treating comminuted intertrochanteric fractures (AO/OTA classification, A2)	Retrospective cohort study	Proximal femoral nail antirotation (PFNA)	Compression hip screw (CHS)
Taqi 2021^125^	Pakistan	To compare the outcomes between proximal femoral locking plate and proximal femoral nail in unstable pertrochanteric fracture, Boyd & Griffin type III/IV	Prospective cross-sectional study	Proximal femoral nail (PFN)	Proximal femoral locking compress plate (PF-LCP)
Tucker 2018^a^ ^126^	United Kingdom - Northern Ireland	To evaluate the functional outcomes, revision, and mortality rates of 3 implants used for unstable intertrochanteric hip fractures; the sliding hip screw (SHS), with or without a trochanteric stabilization plate (TSP); and a cephalomedullary nail (CMN)	Prospective cohort study	Cephalomedullary nail (CMN)	Sliding hip screw (SHS) + trochanteric stabilization plate (TSP)
Utrilla 2005^a^ ^127^	Spain	To compare the results between a new intramedullary Gamma nail and a compression hip screw in the treatment of trochanteric fractures	RCT	Trochanteric gamma nail (TGN)	Compression hip screw (CHS)
Verettas 2010^128^	Greece	To present a comparative study of these two methods regarding their systemic effects on this group of patients	RCT	Intramedullary (IM) nail	Dynamic hip screw (DHS) and plate
Wang 2019^129^	China	To compare effect of proximal femoral nail antirotation (PFNA) and dynamic hip screw (DHS) internal fixation on serum inflammatory mediators (CRP, IL-1, IL-6 and TNF-alpha), myocardial injury markers (cTnT, CK-MB), and Myo-heart failure marker (BNP) in elderly patients with intertrochanteric fractures	RCT	Proximal femoral nail (PFN)	Dynamic hip srew (DHS)
Warren 2020^130^	United States	To compare early postoperative outcomes and complications in patients treated with SHS to those treated with CMN in IT and BC hip fractures	Propensity-matched study	Cephalomedullary nail (CMN)	Sliding hip screw (SHS)
Whale 2016^131^	United States	To compare failure and medical complications of intertrochanteric femoral fractures repaired by CMN or SHS	Retrospective cohort study	Cephalomedullary nail (CMN)	Sliding hip screw (SHS)
Wolf 2022^a^ ^132^	Sweden	To compares the risk of death after the use of SHS or IMN in patients with fractures amenable to both treatment types, i.e., the more stable A1 and the less stable A2-type THF	Prospective cohort study	Intramedullary (IM) nail	Sliding hip screw (SHS)
Xu 2010^a^ ^133^	China	To compare the outcome of elderly patients with an unstable pertrochanteric fracture, treated with a proximal femoral nail antirotation device (PFNA) or a dynamic hip screw (DHS)	RCT	Proximal femoral nail antirotation (PFNA)	Dynamic hip screw (DHS)
Yamauchi 2014^134^	Japan	To compare the differences in functional recovery between patients undergoing plate and nail fixation in the very early period after surgery	Retrospective non-randomized study	Proximal femoral nail (PFN)	Dynamic hip srew (DHS)
Yeganeh 2016^135^	Iran	To compare and examine the consequences of the using intramedullary nailing method versus Dynamic Hip Screw	Prospective cohort study	Proximal femoral nail (PFN)	Dynamic hip srew (DHS)
Yu 2016a^136^	China	To evaluate whether PFNA-II (Asia proximal femoral nail anti-rotation) and DHS (dynamic hip screw) carry substantial post-operative hidden blood loss and to compare PFNA-II with DHS in terms of post-operative hidden blood loss in elderly high-risk patients with intertrochanteric femur fractures(IFFs)	Retrospective cohort study	Proximal femoral nail antirotation (PFNA-III)	Dynamic hip srew (DHS)
Yu 2016b^a^ ^137^	China	To compare DHSs with PFNAs in the management of stable intertrochanteric fractures	Retrospective RCT	Proximal femoral nail antirotation (PFNA)	Dynamic hip srew (DHS)
Zang 2018^138^	China	To compare the clinical efficacy of three different internal fixation methods, i.e. proximal femoral locking compress plate (PF-LCP), proximal femoral nail antirotation (PFNA) and dynamic hip screw (DHS) system in intertrochanteric femur fracture	Prospective comparative study	Proximal femoral nail antirotation (PFNA)	Dynamic hip screw (DHS)
Zehir 2015^139^	Turkey	To determine whether intramedullary fixation with proximal femoral nail antirotation produces comparable outcomes to dynamic hip screw in the treatment of unstable trochanteric fractures	RCT	Proximal femoral nail antirotation (PFNA)	Dynamic hip screw (DHS)
Zeng 2017^a^ ^140^	China	To evaluate long-term radiographic and functional outcomes between dynamic hip screw (DHS) and proximal femoral nail antirotation (PFNA) fixation for treatment of osteoporotic type 31-A1 intertrochanteric femoral fractures (IFFs) among elderly patients	Retrospective cohort study	Proximal femoral nail antirotation (PFNA)	Dynamic hip screw (DHS)
Ziran 2009^141^	United States	To compare the postoperative rehabilitation in patients with peritrochanteric fractures treated with a compression hip screw or an intramedullary hip screw	Prospective cohort study	Intramedullary hip screw (IMHS)	Compression hip screw (CHS)
Zou 2009^142^	China	To compare functional outcome and complications of the PFNA device with those of a traditional extramedullary device, the dynamic hip screw (DHS), in patients with trochanteric fracture	RCT	Proximal femoral nail antirotation (PFNA)	Dynamic hip screw (DHS)

^a^Studies included in the meta-analysis (n = 42 studies).

### Quality Assessment

**Figure 2.**
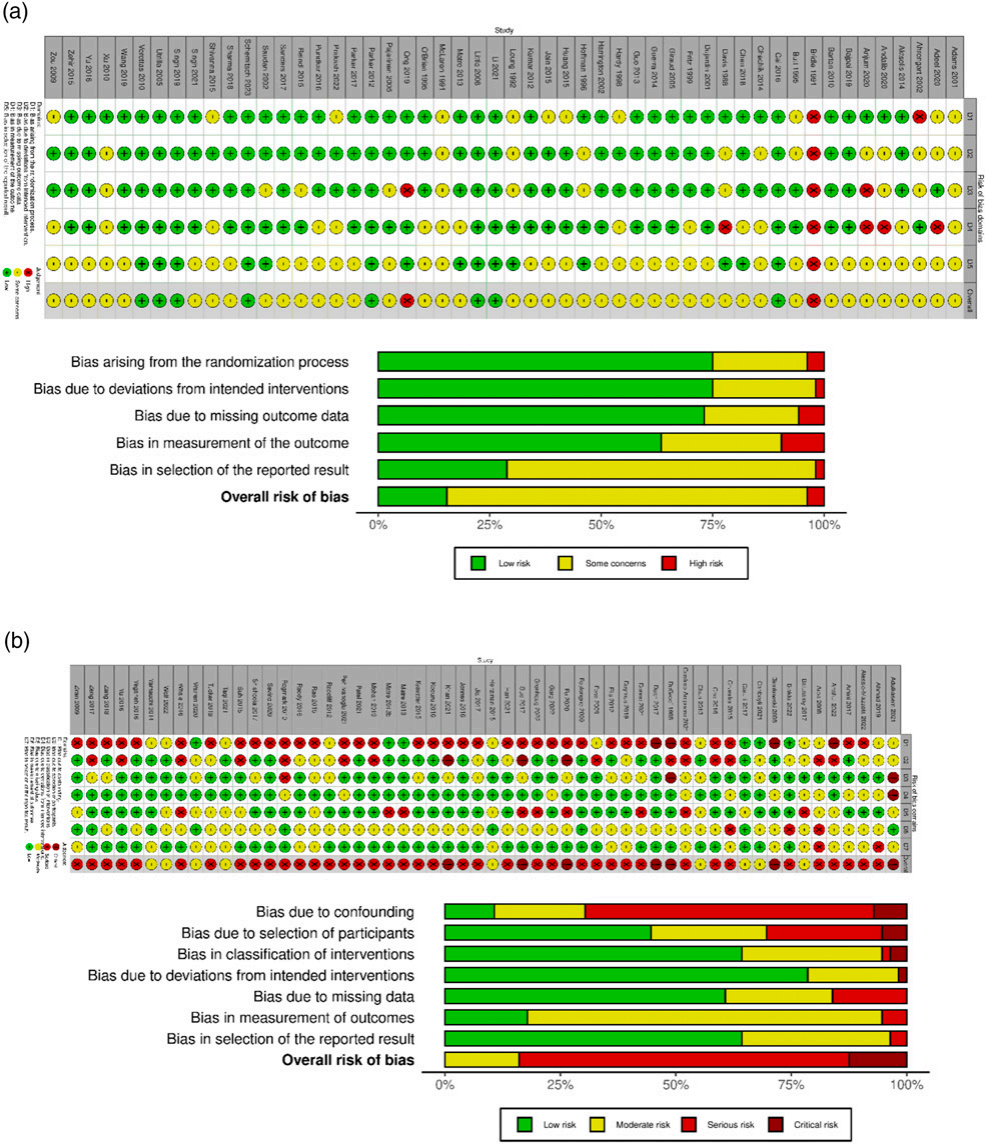
(A) Risk of Bias Summary for RCTs, (B) Risk of Bias for Non-randomized studies

The data bias assessment between the 52 RCTs and the 56 non-randomized studies demonstrated notable differences in methodological rigor and potential biases. In the RCTs, most of the studies (42 out of 52) were classified as having some concerns, indicating minor methodological limitations that may influence the reliability of their findings. However, a noteworthy proportion of the RCTs (8 out of 52) were deemed to have a low risk of bias, indicating robust methodological practices and providing confidence in their results’ validity. Conversely, only 2 studies were categorized as having a high risk of bias, signifying significant methodological shortcomings that may compromise the trustworthiness of their outcomes (Figure 2A).

The risk of bias assessment for the non-randomized studies revealed more concerning findings. Among these studies, 7 were identified as having a critical risk of bias, indicating significant methodological limitations that may affect the validity of their results. Furthermore, a notable portion of the non-randomized studies (40 out of 56) were classified as having a serious risk of bias, suggesting substantial concerns regarding the study designs and methodologies employed. Also, 9 studies were deemed to have a moderate risk of bias, indicating methodological limitations that could impact their reliability (Figure 2B).

While both RCTs and non-randomized studies were subject to risk of bias assessments, the RCTs exhibited better methodological rigor, with a lower proportion of studies categorized as having a high risk of bias. Conversely, the non-randomized studies had a higher prevalence of studies with serious and critical risk of bias, indicating more substantial methodological limitations. Most studies included in the meta-analysis presented some risk of bias.

### Meta-Analysis of Outcomes at Last Follow-Up (12 Months)

In the meta-analysis, outcomes of 42 studies were included.

### Functional Independence

**Figure 3.**
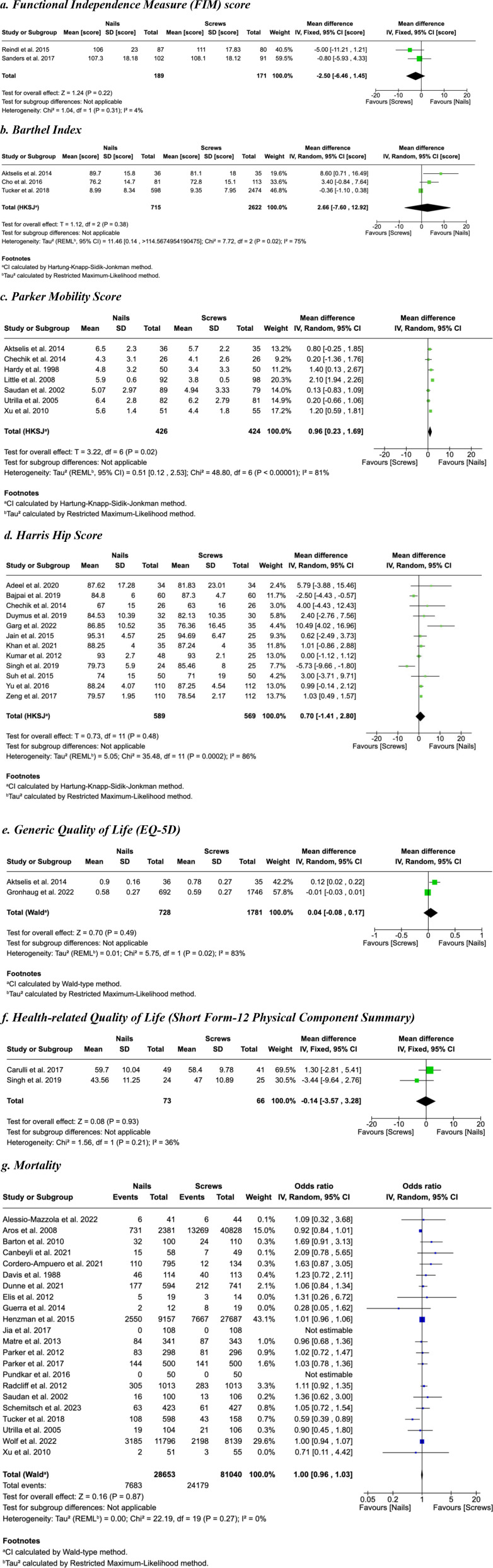
Forest Plots. (A) Functional Independence Measure (FIM) score, (B) Barthel Index, (C) Parker Mobility Score, (D) Harris Hip Score, (E) Generic Quality of Life (EQ-5D), (F) Health-related Quality of Life (Short Form-12 Physical Component Summary)

The pooled estimate for the Functional Independence Measure (FIM), combining the results of both studies using the fixed effect model had no statistically significant difference between the two interventions (mean difference (MD) = −2.50, 95% CI [−6.46, 1.45], Z = 1.24, *P* = 0.22) (Figure 3A). The MD of the three studies for the Barthel Index score was 2.66, 95% CI [−7.60, 12.92], T = 1,12, df = 2, *P* = 0.38) using the random effect model (Figure 3B). We conducted a sensitivity analysis by excluding Tucker et al,^126^ then the remaining studies were homogeneous (I^2^ = 23%). The results showed a MD of 4.89, 95% CI [−25.00, 34.79], T = 2.08, df = 1, *P* = 0.29. The MD of the seven studies for the Parker mobility score was 0.96, 95% CI [0.23, 1.69], T = 3.22, df = 6, *P* = 0.02 using the random effects model, indicating a slight preference for the nails (Figure 3C). The sensitivity analysis excluding Little et al,^95^ shows homogeneous (I^2^ = 31%). Similarly, the results showed a modest preference for the nails (MD = 0.70, 95% CI [0.12, 1.28], T = 3.11, df = 5, *P* = 0.03). The MD for the Harris hip score was 0.70, 95% CI [−1.41, 2.80], T = 0.73, df = 11, *P* = 0.48. We conducted a sensitivity analysis by removing Bajpai et al^47^ and Singh et al,^123^ which showed homogeneous amongst the remaining studies (I^2^ = 0%). The results showed a slight preference for the nails (MD = 0.94, 95% CI [0.34, 1.54], T = 3.54, df = 9, *P* = 0.006) (Figure 3D).

### Quality of Life

The MD for the two studies reporting on the generic quality of life (EQ-5D) measure was 0.04, 95% CI [−0.08, 0.17], Z = 0.70, *P* = 0.49, using the random effect model, indicating no difference between the two intervention groups (Figure 3E). Similarly, the MD for the health-related quality of life (Short Form-12 Physical Component Summary) was −0.14, 95% CI [−3.57, 3.28], Z = 0.08, *P* = 0.93, showing no difference between groups (Figure 3F).

### Mortality

The odds ratio (OR) for mortality at 12 months was 1.00, 95% CI [0.96, 1.03], Z = 0.16, *P* = 0.87, suggesting no statistically significant difference between the two intervention groups (Figure 3G).

### Outcomes Not Included in Meta-Analysis

Findings from studies (n = 81) with insufficient data (ie, missing means, or standard deviations) were excluded from the meta-analysis. The outcomes were divided into seven subcategories. 19 studies reported on functional outcomes (Kyle’s Criterion, Functional Recovery Score of Zuckerman, Salvati and Wilson Score, Koval Score, Lower Extremity Measure (LEM), Sahlstrand grading, etc.), with 6/8 studies having a significant result in favor of the IMN and 2/8 studies in favor of the DHS. Two studies reported on movement (sitting in a wheelchair, five-fold-sit-to-stand test time), with 2 significant results, both favoring the IMN. Two studies reported on the place of care post-discharge, both with non-significant results. Two studies reported on hip fracture related readmissions with only one study with a significant result favoring the DHS. A total of 29 studies reported on walking /mobility /ambulation outcomes (ie, Koval’s grade, ambulatory independence score, able to walk 15-meter test, recovery of previous walking ability, 2-min walking test, Merle D’Aubigne Score Deficit, etc.) with 9 studies reporting significant results, 9/10 studies favoring the IMN. 10 studies reported on weightbearing status, with 3/4 studies reporting significant results favoring the IMN. 19 studies reported on the Harris hip score, with 4/6 studies significant results favoring the IMN. 10 studies reported on mortality and 3 studies on the generic quality of life with no significant results. Further details on the outcomes are available in Table 2.

**Table 2.** Outcomes not Included in the Meta-Analysis

Author	Country	Study design	Intervention 1: Nails	Intervention 2: Screws	Outcomes	Results	Interpretation	Results timeframe
Functional outcomes								
Adulkasem 2021^37^	Thailand	Retrospective cohort study	Proximal femoral nail antirotation (PFNA)	Dynamic hip screw (DHS)	Return to preinjury functional status	mOR = 0.54 CI = 0.19–1.55 *P* = 0.253	NS	12 months
Andalib 2020^43^	Iran	RCT	Cephalomedullary nails	Dynamic hip screw (DHS)	Return to previous activity (%)	I1 = 18.4 (n = 38) I2 = 81.81 (n = 55) *P* < 0.001	S (nail < **screw**)	>6 months
Anjum 2020^44^	Pakistan	RCT	Proximal femoral nail (PFN)	Dynamic hip screw (DHS)	Kyle’s Criterion (Good to excellent) (%)	I1 = 98.66 (n = 75) I2 = 92 (n = 75) *P* = 0.050	S (**nail** > screw)	6 months
Cai 2016^54^	China	RCT	Intramedullary fixation	Extramedullary fixation	Functional Recovery Score of Zuckerman (mean ± SD)	I1 = 36.10 ± 2.38 (n = 106) I2 = 35.96 ± 1.99 (n = 92) *P* = 0.64	NS	12 months
Chechik 2014^58^	Israel	RCT	Expandable proximal femoral nail (EPFN)	Dynamic hip screw (DHS)	Change of independence (%)	I1 = 55 (n = 26) I2 = 65 (n = 26) *P*>0.05	NS	12 months
Chen 2018^59^	China	RCT	Proximal femoral nail antirotation (PFNA)	Dynamic hip srew (DHS)	Sanders excellent and good rate (%)	I1 = 83.3 (n = 18) I2 = 50 (n = 18) *P* = 0.037	S (**nail** > screw)	6 months
Duan 2017^65^	China	Prospective cohort study	Proximal femoral nail antirotation (PFNA)	Dynamic hip srew (DHS)	Acceptance rate of postoperative functional recovery (%)	I1 = 92 (n = 46) I2 = 81 (n = 62) *P* < 0.05	S (**nail** > screw)	9-18 months
Dujardin 2001^66^	France	RCT	Nail	Trochanteric hip screw (THS)	Salvati and Wilson score (mean ± SD)	I1 = 7.8 ± 2.1 (n = 30) I2 = 8.7±1 (n = 30) *P* < 0.05	S (nail < **screw**)	6 months
Guerra 2014^77^	Brazil	RCT	Proximal femoral nail (PFN)	Dynamic hip screw (DHS)	Zuckerman Functional Recovery Score (mean ± SD)	I1 = 33.80±7.11 (n = 12) I2 = 35.09 ± 9.61 (n = 19) *P* = 0.468	NS	12 months
Guo 2013^78^	China	RCT	Proximal femoral nail antirotation (PFNA)	Percutaneous compression plate (PCCP)	Oxford hip score (OHS) (mean ± SD)	I1 = 87.6±8.4(n = 45) I2 = 88.4 ± 9.0 (n = 45) *P* = 0.6640	NS	12 months
Koduru 2016^90^	India	Prospective cohort study	Proximal femoral nail (PFN)	Dynamic hip screw (DHS)	Good-to-excellent functional outcome (%)	I1 = 95 (n = 20) I2 = 50 (n = 20)	Not applicable	12 months
Kyavater 2015^92^	India	Prospective cohort study	Proximal femoral nail (PFN)	Dynamic hip screw (DHS)	Excellent function outcome results (%)	I1 = 67.64 (n = 34) I2 = 50 (n = 34)	Not applicable	not reported
Patel 2021^106^	India	Prospective cohort study	Proximal femoral nail (PFN)	Dynamic hip screw (DHS)	Excellent functional outcome in stable fractures (%) Excellent functional outcome in unstable fractures (%)	I21 = 25 (n = 12) I2 = 25 (n = 16) *P* = 0.97 I1 = 30.7 (n = 13) I12 = 11.1 (n = 9) *P* = 0.04	NS S (**nail**>screw)	12 months 12 months
Pundkar 2016^109^	India	RCT	Proximal femoral nail (PFN)	Dynamic hip screw (DHS)	Kyles criteria (“Excellent”)	I1 = 20 (40%, n = 50) I2 = 16 (32%, n = 50)	Not applicable	12 months
Sanders 2017^115^	Canada	RCT	InterTAN intramedullary device (IT)	Sliding hip screw (SHS)	Low extremity measure (LEM) (mean ± SD)	I1 = 63.1 ± 2.4 (n = 123) I2 = 63.4 ± 2.6 (n = 126) *P* = 0.94	NS	12 months
Shivanna 2015^121^	India	RCT	Proximal femoral nail (PFN)	Dynamic hip screw (DHS)	Functional outcome - sahlstrand grading (Excellent results)	I1: 6 *P*atients (n = 15, 40.00%) I2: 3 *P*atients (n = 15, 20.0%) *P* < 0.037	S (**nail** > screw)	4 months
Suh 2015^124^	Korea	Retrospective cohort study	Proximal femoral nail antirotation (PFNA)	Compression hip screw (CHS)	Koval score (mean ± SD)	I1 = 74±15 (n = 50) I2 = 71 ± 19 (n = 50) *P* = 0.758	NS	12 months
Yamauchi 2014^134^	Japan	Retrospective non-randomized study	Proximal femoral nail (PFN)	Dynamic hip screw (DHS)	Recovery of activities of daily living (ADLs) according to JOA hip functional scores	I1 = 16.30 (SD 6.85, n = 10) I2 = 9.50 (SD 5.18, n = 8) *P* = 0.027	S (**nail** > screw)	1 month
Zou 2009^142^	China	RCT	Proximal femoral nail antirotation (PFNA)	Dynamic hip screw (DHS)	Salvati and Wilson scores (Excellent, >32)	I1 = 38 (n = 58) I2 = 36 (n = 63)	Not applicable	12 months
Movement								
Foulongne 2009^71^	France	Case-control study	Mini-invasive nailing device (BCM nail)	Dynamic hip screw (DHS)	Sitting in a wheelchair (mean days ± SD)	I1 = 4±1.44 (n = 30) I2 = 4.76 ± 1.53 (n = 30) *P* < 0.03	S (**nail** > screw)	not reported
Li 2021^94^	China	RCT	Proximal femoral nail antirotation (PFNA)	Dynamic hip screw (DHS)	Five-fold-sit-to stand test time (s ± SD)	I1 = 51.3±11.0 (n = 40) I2 = 73.6 ± 13.0 (n = 40) *P* = 0.00	S (**nail** > screw)	18 months
Place of care								
Saudan 2002^116^	Switzerland	RCT	Proximal femoral nail (PFN)	Dynamic hip screw (DHS)	Habitation-home (#)	I1 = 37(n = 79) I2 = 50(n = 89) *P* = 0.22	NS	12 months
Saudan 2002	Switzerland	RCT	Proximal femoral nail (PFN)	Dynamic hip screw (DHS)	Habitation-nursing home (#)	I1 = 42(n = 79) I2 = 39(n = 89) *P* = 0.22	NS	12 months
Ziran 2009^141^	United States	Prospective cohort study	Intramedullary hip screw (IMHS)	Compression hip screw (CHS)	Disposition (%) Home	I1 = 18(n = 49) I2 = 20(n = 45) *P* = NS	NS	Not reported
Ziran 2009	United States	Prospective cohort study	Intramedullary hip screw (IMHS)	Compression hip screw (CHS)	Disposition (%) Rehabilitation	I1 = 51(n = 49) I2 = 29(n = 45) *P* = 0.07	NS	Not reported
Ziran 2009	United States	Prospective cohort study	Intramedullary hip screw (IMHS)	Compression hip screw (CHS)	Disposition (%) Skilled care	I1 = 31(n = 49) I2 = 51(n = 45) *P* = 0.07	NS	Not reported
Readmissions related to the hip fracture								
Rogmark 2010^114^	Sweden	Retrospective cohort study	Intramedullary (IM) nail	Sliding hip screw (SHS)	Re-admitted pertrochanteric fractures (%)	I1 = 28 (n = 6041) I2 = 28 (n = 6429) *P* = 0.639	NS	6 months
Rogmark 2010	Sweden	Retrospective cohort study	Intramedullary (IM) nail	Sliding hip screw (SHS)	Re-admitted subtrochanteric fracture (%)	I1 = 28 (n = 2040) I2 = 28 (n = 475) *P* = 0.788	NS	6 months
Rogmark 2010	Sweden	Retrospective cohort study	Intramedullary (IM) nail	Sliding hip screw (SHS)	Readmission due to hip complications pertrochanteric fractures (%)	I1 = 5.1 (n = 6041) I2 = 3.8 (n = 6429) *P* < 0.001	S (nail < **screw**)	6 months
Rogmark 2010	Sweden	Retrospective cohort study	Intramedullary (IM) nail	Sliding hip screw (SHS)	Readmission due to hip complications subtrochanteric fracture (%)	I1 = 6.3 (n = 2040) I2 = 6.1 (n = 475) *P* = 0.86	NS	6 months
Warren 2020^130^	United States	Propensity-matched study	Cephalomedullary nail (CMN)	Sliding hip screw (SHS)	Readmission	I1 = 580 (7.4%, n = 8505) I2 = 602 (7.5%, n = 8505) *P* = 0.702	NS	1 month
Walking/mobility/ambulation								
Ahrengart 2002^39^	Sweden	RCT	Gamma nail	Compression hip screw (CHS)	Need walking aid (%)	I1 = 71% (n = 169) I2 = 70% (n = 179)	Not applicable	6 months
Bienkowski 2006^51^	Canada	Prospective cohort study	Trochanteric fixation nail (TFN)	Dynamic hip screw (DHS)	Return to ambulation (%)	I2 = 0.71 (n = 30) I2 = 0.33 (n = 30) *P* = 0.09	NS	6 months
Carulli 2017^56^	Italy	Prospective cohort study	Proximal femoral nail antirotation (PFNA)	Sliding hip screw (SHS)	Independent walking ability (#)	I1 = 58/69 (81.60%) I2 = 45/67 (67.16%) *P* < 0.05	S (**nail** > screw)	3 months
Carulli 2017	Italy	Prospective cohort study	Proximal femoral nail antirotation (PFNA)	Sliding hip screw (SHS)	Restore walking activity and health status pre-fracture (#)	I1 = 49/66 (74.24%) I2 = 41/62 (66.12%) *P*>0.05	NS	12 months
Chechik 2014^58^	Israel	RCT	Expandable proximal femoral nail (EPFN)	Dynamic hip screw (DHS)	Decrease in ambulation score (%)	I1 = 52 (n = 26) I2 = 58 (n = 26) *P* > 0.05	NS	12 months
Cho 2016^60^	Korea	Retrospective cohort study	Proximal femoral nail antirotation (PFNA)	Dynamic hip screw (DHS)	Koval’s grade - maintain (%) - walking ability score	I1 = 56.8 (n = 81) I2 = 50.4 (n = 113) *P* = 0.815	NS	not reported
Cho 2016	Korea	Retrospective cohort study	Proximal femoral nail antirotation (PFNA)	Dynamic hip screw (DHS)	Koval’s grade - 1 grade down (#)	I1 = 25 (n = 81) I2 = 41 (n = 113) *P* = 0.815	NS	not reported
Cho 2016	Korea	Retrospective cohort study	Proximal femoral nail antirotation (PFNA)	Dynamic hip screw (DHS)	Koval’s grade - 2 grade down (#)	I1 = 8 (n = 81) I2 = 11 (n = 113) *P* = 0.815	NS	not reported
Cho 2016	Korea	Retrospective cohort study	Proximal femoral nail antirotation (PFNA)	Dynamic hip screw (DHS)	Koval’s grade - 3 grade down (#)	I1 = 2 (n = 81) I2 = 4 (n = 113) *P* = 0.815	NS	not reported
Chua 2013^61^	Singapore	Retrospective cohort study	Proximal femoral nail antirotation (PFNA)	Dynamic hip screw (DHS)	Ambulatory independence score	I1 = 5 (n = 32) I2 = 4 (n = 38) *P* = 0.001	S (**nail** > screw)	12 months
Cordero-Ampuero 2021^62^	Spain	Retrospective cohort study	Trochanteric nails	Sliding hip screw (SHS)	Recovery of previous walking ability	I1 = 261 (n = 764) I2 (n = 129)	Not applicable	12 months
Davis 1988^63^	United Kingdom	RCT	Kuntscher-Y nail (K-Y)	Sliding hip screw (SHS)	Failed to regain their prefracture level of walking ability (#)	I1 = 30 (n = 58) I2 = 26 (n = 62)	Not applicable	12 months
Dujardin 2001^66^	France	RCT	Nail	Trochanteric hip screw (THS)	Painless mobilization (wk) (mean ± SD)	I1 = 4.3±1.3 (n = 30) I2 = 8.2 ± 3.7 (n = 30) *P* < 0.001	S (**nail** > screw)	not reported
Foss 2009^70^	Denmark	Prospective cohort study	Intramedullary hip screws (IMHS)	Dynamic hip screw (DHS)	Cumulated ambulation score (mean)	I1 = 9 (n = 15) I2 = 9 (n = 49) *P* = 0.36	NS	not reported
Fritz 1999^72^	Germany	RCT	Gamma nail	Dynamic hip screw (DHS)	Merle d'Aubigne Score Deficit - post-op pain (%) - Deficit means comparison to pretraumatic situation	I1 = 17 (n = 40) I2 = 13 (n = 17)	Not applicable	6 months
Fritz 1999	Germany	RCT	Gamma nail	Dynamic hip screw (DHS)	Merle d'Aubigne Score Deficit - post-op walking (%)	I1 = 45 (n = 40) I2 = 47 (n = 17)	Not applicable	6 months
Fritz 1999	Germany	RCT	Gamma nail	Dynamic hip screw (DHS)	Merle d'Aubigne Score Deficit- post-op mobility (%)	I1 = 18 (n = 40) I2 = 15 (n = 17)	Not applicable	6 months
Fritz 1999	Germany	RCT	Gamma nail	Dynamic hip screw (DHS)	Merle d'Aubigne Score Deficit - post-op total (%)	I1 = 26 (n = 40) I2 = 23 (n = 17)	Not applicable	6 months
Guo 2013^78^	China	RCT	Proximal femoral nail antirotation (PFNA)	Percutaneous compression plate (PCCP)	Walking ability score (mean ± SD)	I1 = 6.7 ± 2.8(n = 45) I2 = 6.9 ± 1.5 (n = 45) *P* = 0.6738	NS	12 months
Guo 2013	China	RCT	Proximal femoral nail antirotation (PFNA)	Percutaneous compression plate (PCCP)	Recovery of walking ability to pre-injury status - Yes (#)	I1 = 27 (n = 45) I2 = 26 (n = 45) *P* = 1.00	NS	12 months
Harrington 2002^82^	United Kingdom	RCT	Intramedullary hip screw (IMHS)	Compression hip screw	Ambulatory status (# of patients retaining pre-injury living status)	I1 = 19 (n = 30) I2 = 22 (n = 33)	Not applicable	12 months
Koduru 2016^90^	India	Prospective cohort study	Proximal femoral nail (PFN)	Dynamic hip screw (DHS)	Regain pre-injury walking ability (#)	I1 = 14 ( = 20) I2 = 5 (n = 20) *P* = 0.07	NS	3 months
Li 2021^94^	China	RCT	Proximal femoral nail antirotation (PFNA)	Dynamic hip screw (DHS)	10-meter walking speed (m/s ± SD)	I1 = 1.6 ± 0.2 (n = 40) I2 = 0.9 ± 0.1 (n-40) *P* = 0.00	S (**nail** > screw)	18 months
Little 2008^95^	United Kingdom	RCT	Holland nail	Dynamic hip screw (DHS)	Patients with mobility restored (%)	I1 = 64 (n = 92) I2 = 37 (n = 98) *P* < 0.001	S (**nail** > screw)	12 months
McLaren 1991^99^	United Kingdom	RCT	Pugh nail	Dynamic hip screw (DHS)	Walking ability - frame (%)	I1 = 57 (n = 40) I2 = 55 (n = 44)	Not applicable	6 months
Pajarinen 2005^103^	Finland	RCT	Proximal femoral nail (PFN)	Dynamic hip screw (DHS)	Restoration of walking ability (%)	I1 = 76.2 (n = 42) I2 = 53.7 (n = 41) *P* = 0.040	S (**nail** > screw)	4 months
Parker 2017^105^	United Kingdom	RCT	Intramedullary (IM) nail	Sliding hip screw (SHS)	Mean change in mobility scale (mean ± SD)	I1 = 0.5± 2.0 (n = 348) I2 = 1.0 ± 2.0 (n = 350) *P* = 0.001	S (**nail** > screw)	12 months
Sanders 2017^115^	Canada	RCT	InterTAN intramedullary device (IT)	Sliding hip screw (SHS)	2 min walking test - 2 MWT (%)	I1 = 83 (n = 123) I2 = 80 (n = 126) *P* = 0.35	NS	12 months
Verettas 2010^128^	Greece	RCT	Intramedullary (IM) nail	Dynamic hip screw (DHS) and plate	Independent walking (Mean days ± SD)	I1 = 7.4 ± 2.6 (n = 59) I2 = 8.2 ± 2.9 (n = 59) *P* = 0.117	NS	Not reported
Xu 2010^133^	China	RCT	Proximal femoral nail antirotation (PFNA)	Dynamic hip screw (DHS)	Recovery of walking ability to pre-operative status	I1 = 27/40 (67.5%, n = 51) I2 = 19/43 (44.2%, n = 55) *P* = 0.033	S (**nail** > screw)	12 months
Yeganeh 2016^135^	Iran	Prospective cohort study	Proximal femoral nail (PFN)	Dynamic hip screw (DHS)	Walking recovery and doing daily activities (%)	I1 = 62 (n = 60) I2 = 22 (n = 54)	Not applicable	3 months
Leung 1992^93^	United Kingdom	RCT	Gamma nail	Dynamic hip screw (DHS)	Mobility-independent (#)	I1 = 34 (n = 93) I2 = 31(n = 93) *P*>0.05	NS	6 months
Ong 2019^102^	United Kingdom	RCT	Cephalomedullary nail (CMN)	Sliding hip screw (SHS)	Mobility score ≥7 (mean ± SD)	I1 = 1.16 ± 1.60 (n = 188) I2 = 1.75 ± 1.98 (n = 192) *P* = 0.0015	S (nail < **screw**)	12 months
Mohan 2019^100^	India	Retrospective cohort study	Proximal femoral nail (PFN)	Dynamic hip screw (DHS)	Parker mobility score (mean ± SD)	I1 = 7.6 ± 1.7 I2 = 8 ± 0.72 *P* = 0.5686	NS	6 months
Shishodia 2017^120^	India	Prospective cohort study	Proximal femoral nail (PFN)	Dynamic hip screw (DHS)	Walking ability (Parker & Palmer mobility score)	I1: 8.60 ± 0.74 (n = 15) I2: 8.93 ± 0.26 (n = 15) *P* = 0.10	NS	9 months
Matre 2013c^98^	Norway	RCT	Trochanteric antegrade nail (InterTan)	Sliding hip screw (SHS)	Up & Go test (mean score in seconds)	I1 = 27 (n = 154) I2 = 25 (n = 160) *P* = 0.60	NS	12 months
Zehir 2015^139^	Turkey	RCT	Proximal femoral nail antirotation (PFNA)	Dynamic hip screw (DHS)	Recovery of walking ability (%)	I1 = 76 (n = 96) I2 = 66.7 (n = 102) *P* = 0.14	NS	6 months
Zehir 2015	Turkey	RCT	Proximal Femoral Nail Antirotation (PFNA)	Dynamic hip screw (DHS)	Unrestricted/independent walking (%)	I1 = 55.2 (n = 96) I2 = 28.4 (n = 102) *P* < 0.001	S (**nail** > screw)	6 months
Weightbearing								
Amini 2017^42^	Not reported	Retrospective cohort study	Cephalomedullary nails	Sliding hip screw (SHS)	Non-weight Bearing (#)	I1 = 4 (n = 16) I2 = 6 (n = 21)	Not applicable	9 months
Anshul 2022^45^	India	Prospective cohort study	Proximal femoral nail (PFN)	Dynamic hip screw (DHS)	Full weight bearing	I1 = 14 (n = 40) I2 = 18 (n = 40)	Not applicable	Not reported
Biakto 2022^50^	Indonesia	Retrospective cross-sectional study	Proximal femoral nail antirotation (PFNA)	Dynamic hip screw (DHS)	Weight-bearing (%)	I1 = 46.2(26) I2 = 61.7(n = 81) *P* = 0.162	NS	not reported
De Groof 1988^64^	Belgium	Retrospective cohort study	Böhler nail	Dynamic hip screw (DHS)	Full weight-bearing (mean days)	I1 = 65.1 (n = 129) I2 = 39.3 (n = 129) *P* < 0.005	S (nail < **screw**)	not reported
Guo 2017^79^	China	Retrospective cohort study	Proximal femoral nail antirotation (PFNA)	Dynamic hip screw (DHS)	Postoperative weightbearing time (days ± SD)	I1 = 7.4 ± 1.3 (n = 82) I2 = 12.5±2.8 (n = 71) *P* = 0.024	S (**nail** > screw)	not reported
Huang 2015^85^	China	RCT	Proximal femoral nail antirotation (PFNA)	Dynamic hip screw (DHS)	Postoperative weight-bearing time (days)	I1: 42.15 ± 3.62 (n = 30) I2: 57.23 ±3.36 (n = 30) *P* < 0.001	S (**nail** > screw)	not reported
Jonnes 2016^88^	India	Prospective cohort study	Proximal femoral nail (PFN)	Dynamic hip screw (DHS)	Full weight bearing walking (average weeks)	I1 = 7.93 (n = 15) I2 = 11.80 (n = 15)	Not applicable	not reported
Rao 2015^111^	India	Prospective cohort study	Proximal femoral nail (PFN)	Dynamic hip screw (DHS)	Mean time for full weight bearing (weeks)	I1 = 10.6 (n = 40) I2 = 14.8 (n = 40)	Not applicable	6 months
Singh 2021^122^	India	RCT	Proximal femoral nail (PFN)	Dynamic hip screw (DHS)	Full weight bearing (mean weeks ± SD)	I1 = 14.20 ± 1.52 (n = 15) I2 = 14.13±1.36 (n = 15) *P* = 0.127	NS	not reported
Wang 2019 ^129^	China	RCT	Proximal femoral nail (PFN)	Dynamic hip screw (DHS)	Weight-bearing (mean weeks ± SD)	I1 = 5.82 ± 1.07 (n = 57) I2 = 7.76±1.45 (n = 57) *P* < 0.001	S (**nail** > screw)	not reported
Harris hip score (not in meta-analysis)								
Adams 2001^35^	United Kingdom	RCT	Gamma nail	Sliding hip screw (SHS)	Harris hip score	I1 = 66.9 (n = 145) I2 = 66.4 (n = 139)	Not applicable	6 months
Ahmad 2019^38^	Parkistan	Prospective cohort study	Intramedullary (IM) nail	Dynamic hip screw (DHS)	Harris hip score	I1 = 88.7 ± 5.6 (n = 3) I2 = 71.7 ± 13.8 (n = 18)	Not applicable	6 months
Balusamy 2017^48^	India	Prospective cohort study	Proximal femoral nail (PFN)	Dynamic hip screw (DHS)	Harris hip score -mean	I1 = 83.57(n = 20) I2 = 80.2(n = 20)	Not applicable	5 months
Chander 2015^57^	India	Prospective cohort study	Proximal femoral nail (PFN)	Dynamic hip screw (DHS)	Harris hip score (mean ± SD)	I1 = 83.7 ± 3.71 (n = 15) I2 = 83 ± 3.89 (n = 15) *P*>0.05	NS	6 months
Giraud 2005^75^	France	RCT	Targon nail	Dynamic hip screw (DHS)	Harris hip score (mean)	I1 = 60 (n = 34) I2 = 59 (n = 26)	Not applicable	3 months
Guo 2017^79^	China	Retrospective cohort study	Proximal Femoral Nail Antirotation (PFNA)	Dynamic Hip Screw (DHS)	Harris score excellence rate (%)	I1 = 93.9 (n = 82) I2 = 83.1 (n = 71) *P* = 0.034	S (**nail** > screw)	not reported
Han 2021^80^	China	Cohort study	Proximal femoral nail antirotation (PFNA)	Dynamic hip screw (DHS)	Harris hip score excellent and good (%)	I1 = 85 (n = 80) I2 = 64 (n = 50) *P* = 0.006	S (**nail** > screw)	not reported
Huang 2015^85^	China	RCT	Proximal femoral nail antirotation (PFNA)	Dynamic hip screw (DHS)	Harris hip score	I1: 93.30% (n = 30) I2: 70% (n = 30) *P* = 0.06	NS	12 months
Jonnes 2016^88^	India	Prospective cohort study	Proximal femoral Nail (PFN)	Dynamic Hip Screw (DHS)	Harris hip score (average)	I1 = 90.33 (n = 15) I2 = 89.08 (n = 15) *P* = 0.31	NS	12 months
Mohan 2019^100^	India	Retrospective cohort study	Proximal femoral nail (PFN)	Dynamic hip screw (DHS)	Modified Harris hip score (mean ± SD)	I1 = 69 ±5.52 I2 = 70.6 ± 4.08*P* = 0.9203	NS	6 months
Prakash 2022^108^	India	RCT	Proximal femoral nail (PFN)	Dynamic hip screw (DHS)	Harris hip score (Mean ± SD)	I1 = 89.26 ± 6.53 (n = 23) I2 = 84.3 ± 7.68 (n = 23) *P* = 0.023	S (**nail** > screw)	6 months
Rao 2015^111^	India	Prospective cohort study	Proximal femoral nail (PFN)	Dynamic hip screw (DHS)	Harris hip score (Excellent)	I1 = 72.2% (n = 40) I2 = 57.8% (n = 40)	Not applicable	6 months
Reddy 2016^112^	India	Prospective cohort study	Reconstruction nail	Dynamic hip screw (DHS)	Harris hip score	I1 = 88.4 (n = 30) I2 = 86.1 (n = 30) *P* = 0.269	NS	9 months
Sevinc 2020^118^	Turkey	Prospective cohort study	Proximal femoral nail antirotation (PFNA)	Dynamic hip screw (DHS)	Harris hip score (mean)	I1 = 66.5 (n = 56) I2 = 80.5 (n = 66) *P* = 0.001	S (nail < s**crew**)	12 months
Sharma 2018^119^	India	RCT	Proximal femoral nail (PFN)	Dynamic hip screw (DHS)	Harris hip score (mean)	I1 = 82.2 (n = 31) I2 = 88.7 (n = 29) *P* < 0.01	S (nail < **screw**)	6 months
Singh 2021^133^	India	RCT	Proximal femoral nail (PFN)	Dynamic hip screw (DHS)	Harris hip score (mean ± SD)	I1 = 82.67 ± 5.98 (n = 15) I2 = 78.93±8.51 (n = 15) *P* = 0.175	NS	not reported
Taqi 2021^125^	Pakistan	Prospective cross sectional study	Proximal femoral nail (PFN)	Proximal Femoral Locking Compress Plate (PF-LCP)	Harris hip score excellent and good (%)	I1: 93.1% (n = 30) I2: 79.3% (n = 29) *P* = 0.06	NS	12 months
Yu 2016a^136^	China	Retrospective cohort study	Proximal femoral nail antirotation (PFNA-III)	Dynamic hip screw (DHS)	Harris hip score (mean ± SD)	I1 = 85.63 ± 4.13 (n = 186) I2 = 82.59 ± 5.01 (n = 177) *P* = 0.000	S (**nail** > screw)	not reported
Zang 2018^138^	China	Prospective comparative study	Proximal femoral nail antirotation (PFNA)	Dynamic hip screw (DHS)	Harris hip score (mean ± SD)	I1 = 83.78 ± 9.32 (n = 50) I2 = 73.31±8.71 (n = 50)	Not applicable	6 months
Mortality (not in meta-analysis)								
Barton 2010^49^	United Kingdom	RCT	Long gamma nail	Sliding hip screw (SHS)	Mortality (#)	I1 = 21 (n = 100) I2 = 11 (n = 110) *P* = 0.13	NS	1 month
Bridle 1991^52^	United Kingdom	RCT	Gamma nail	Dynamic hip screw (DHS)	Deaths (#)	I1 = 15 (n = 49) I2 = 19 (n = 51)	Not applicable	6 months
Butt 1995^53^	England	RCT	Gamma nail	Dynamic hip screw (DHS)	Deaths (#)	I1 = 5(n = 48) I2 = 2(n = 47)	Not applicable	not reported
Giraud 2005^75^	France	RCT	Targon nail	Dynamic hip screw (DHS)	Mortality (#)	I1 = 2 (n = 34) I2 = 1 (n = 26)	Not applicable	3 months
Hoffman 1996^84^	Australia	RCT	Gamma nail	Ambi hip screw	Mortality (#)	I1 = 5 (n = 31) I2 = 3(n = 36) *P* = NS	NS	6 months
Leung 1992^93^	United Kingdom	RCT	Gamma nail	Dynamic hip screw (DHS)	Mortality (#)	I1 = 13 (n = 113) I2 = 15(n = 113)	Not applicable	6 months
O’Brien 1995^101^	Canada	RCT	Gamma nail	Dynamic hip screw (DHS)	Total Mortality (#)	I1 = 15 (n = 53) I2 = 1 (n = 49) *P*>0.05	NS	not reported
Pehlivanoglu 2021^107^	Turkey	Retrospective cohort study	Proximal femoral nail (PFN)	Dynamic hip screw (DHS)	Mortality (%)	I1 = 73.6 (n = 57) I2 = 67.6 (n = 65) *P* = 0.469	NS	not reported
Radcliff 2012^110^	United States	Retrospective cohort study	Intramedullary (IM) Nail	Sliding hip screw (SHS)	Mortality (propensity-score-matched sample) (%)	I1 = 30.1 (n = 1013) I2 = 27.9 (n = 1013) *P* = 0.28	NS	12 months
Whale 2016^131^	United States	Retrospective cohort study	Cephalomedullary nail (CMN)	Sliding Hip Screw (SHS)	Mortality in stable fractures (%)	I1 = 38.1 (n = 84) I2 = 36.1 (n = 97) *P* = 0.376	NS	not reported
Generic quality of life (EQ-5D) (not in meta-analysis)								
Fu 2020^73^	Taiwan	Retrospective cohort study	Proximal femoral nail antirotation (PFNA)	Dynamic hip screw (DHS)	EQ-5D in 31A2 fracture (mean ± SD)	I1 = 0.61 ± 0.10 (n = 70) I2 = 0.59 ± 0.14 (n = 171) *P* = 0.211	NS	10 months +
Matre 2013a^96^	Norway	Prospective cohort study	Intramedullary (IM) nail	Sliding Hip Screw (SHS)	EQ-5D index score	I1 = 0.58 (n = 376) I2 = 0.55 (n = 1097) *P* = 0.06	NS	12 months
Matre 2013b^97^	Norway	Retrospective cohort study	Intramedullary (IM) nail	Sliding Hip Screw (SHS)	EQ-5D index score (mean)	I1 = 0.57 (n = 312) I2 = 0.55 (n = 491) *P* = 0.23	NS	12 months

### Discussion

This systematic review and meta-analysis examined the effectiveness of two surgical interventions, IM nails vs DHS by comparing the outcomes of functional, quality of life, and survival during a 12-month follow-up period. The meta-analysis found no statistically significant differences in functional independence between IMN and DHS based on the FIM and the Barthel index scores. Modest significance was identified during sensitivity analyses for the Parker mobility score and the Harris hip score favoring the IMN. No significant differences were observed on the generic quality of life, health-related quality or mortality at 12 months. The outcomes excluded from the meta-analysis provided additional context. Studies reporting other functional outcomes such as movement, walking/mobility/ambulation, weightbearing status had their overall results favoring the IMN. Studies on place of care, mortality and quality of life reported no significant results.

The IMN and the DHS are both effective treatments to manage intertrochanteric fractures. For most intertrochanteric hip fractures, the DHS has an established history of successful use, demonstrates high reproducibility and is more cost-effective compared to the IMN.^143^ Alternatively, the IMN is biomechanically superior^144^ and inserted using less invasive surgical approaches. When comparing these two implants, the literature shows similar results with failure rates as the main outcome.^145,146^ There may be some advantages to the IMN when treating the “most unstable” patterns.^144^

Given that the IMN is a more expensive implant, its indiscriminate use for all fractures will greatly impact the cost of treating these injuries. The use of the IMN has increased significantly over the past 20 years.^147^ Despite this trend, the success rate of DHS and IMN fixation in both stable and unstable fractures have been similar in several studies.^97,113^ In a prospective study, Grønhaug and colleagues^76^ assessed data from 17 341 patients within the Norwegian Hip Fracture Register over the period of 2013-2019 and found a significantly lower re-operation rate for IMNs, when compared to DHS for unstable fractures (31-A2 and 31-A3). However, for stable fractures (31-A1), there was no difference recorded in the re-operation rate. Although the “success” of surgery can be measured by fracture healing and/or re-operation rate, many other factors must be considered in order evaluate the overall effectiveness of these two implants. Previous research comparing the DHS and IMN for the treatment of intertrochanteric hip fractures found no significant differences in mortality,^15,146,148,149^ re-operation rate,^145,146,148^ length of stay,^145,148^ major complications,^15,146,149^ nonunion,^15^ infection,^15,145^ mean surgical time,^145^ time to healing,^145^ and failure of fixation rates.^145,146^ However, patients with IMN fixation have demonstrated slightly less blood loss compared to those with DHS.^145,146,149^

This systematic review reported on functional, quality of life, and mortality outcomes found in the published literature. In addition to the meta-analysis, we included a narrative synthesis of the additional outcomes to avoid biased reporting. We found no studies looking at rehabilitation outcomes such as (eg, time to initiation of rehabilitation, rehabilitation length of stay, rehabilitation intensity/frequency, or type of rehabilitation). Further primary studies are needed to look at the effectiveness of DHS and IMN on rehabilitation outcomes for this hip fracture population.

The comparison between DHS and IMN has significant implications for policy, practice, and further research in hip fracture management. Orthopedic surgeons face the challenge of selecting the most appropriate surgical technique based on patient characteristics, fracture type, and institutional resources. While our review indicated some disparity in functional outcomes between the two methods, it is important for clinicians to weigh the comparative effectiveness data and clinical judgment when choosing between DHS and IMN for treatment. When considering research in the treatment of intertrochanteric hip fractures, it is imperative to further understand the nuances in outcomes associated with functional recovery and quality of life between DHS and IMN procedures. RCTs with larger sample sizes and longer follow-up periods, as well as prospective, multicenter studies, are needed to compare these techniques’ effectiveness in diverse patient populations. So far, research has focused more commonly on perioperative outcomes and complications and the there is a lack of research on comparing the recovery and rehabilitation needs for these patients. By ensuring that surgeons have access to the necessary equipment and training, policymakers can contribute to enhanced patient outcomes and long-term reductions in healthcare costs.^12^

### Strengths and Limitations

The strengths of this review include the prospective registration of the study protocol and a comprehensive search of the literature. It also incorporates a meta-analysis to assess the select outcomes. Nonetheless, it is important to acknowledge the limitations. The different 31A fractures groups and subgroups were not analyzed individually, which limited the detection of specific differences in outcomes related to fracture stability. Also, our review did not specifically exclude studies comparing IMN and DHS fixations in patients with reverse obliquity fractures (AO/OTA type A3) although some clinical guidelines^150,151^ recommend the specific use of IMN fixations for this more unstable pattern. Additionally, studies often did not report postoperative radiological outcomes to assess the accuracy of the surgical procedure, which may have impacted patient outcomes. Given that several studies included in our review had methodological limitations, the results should be interpreted with caution. Future studies should address these gaps and consider including postoperative radiological results to improve the comparison and evaluation of these two fixations.

## Conclusion

Overall, our meta-analysis found some differences between the two fixations (IMN vs DHS) for the treatment of intertrochanteric hip fractures. Our meta-analysis revealed that IMN had modest advantages over DHS for functional outcomes. In addition, in our narrative synthesis, the IMN provided some positive results for functional outcomes. Further research on the effect of these fixations on functional rehabilitation outcomes is needed.

## Supplemental Material

Supplemental Material - Comparing Intramedullary Nails versus Dynamic Hip Screws in the Treatment of Intertrochanteric Hip Fractures on Post-operative Rehabilitation Outcomes – A Systematic Review and Meta-Analysis Supplemental Material for Comparing Intramedullary Nails versus Dynamic Hip Screws in the Treatment of Intertrochanteric Hip Fractures on Post-operative Rehabilitation Outcomes – A Systematic Review and Meta-Analysis by Chantal Backman, RN, MHA, PhD, Ashley Lam, Rosie Papp, Aurelie Tonjock Kolle, Franciely Daina Engel, Wenshan Li, Soha Shah, Colleen Webber, Peter Tanuseputro, Marie-Cecile Domecq, and Steve Papp in Geriatric Orthopaedic Surgery & Rehabilitation

Supplemental Material - Comparing Intramedullary Nails versus Dynamic Hip Screws in the Treatment of Intertrochanteric Hip Fractures on Post-operative Rehabilitation Outcomes – A Systematic Review and Meta-Analysis Supplemental Material for Comparing Intramedullary Nails versus Dynamic Hip Screws in the Treatment of Intertrochanteric Hip Fractures on Post-operative Rehabilitation Outcomes – A Systematic Review and Meta-Analysis by Chantal Backman, RN, MHA, PhD, Ashley Lam, Rosie Papp, Aurelie Tonjock Kolle, Franciely Daina Engel, Wenshan Li, Soha Shah, Colleen Webber, Peter Tanuseputro, Marie-Cecile Domecq, and Steve Papp in Geriatric Orthopaedic Surgery & Rehabilitation

## Data Availability

Data is provided within the manuscript or supplementary information files.*

## References

[1] Dong Y Zhang Y Song K Kang H Ye D Li F . What was the epidemiology and global burden of disease of hip fractures from 1990 to 2019? Results from and additional analysis of the global burden of disease study 2019. Clin Orthop. 2023;481(6):1209-1220. doi:10.1097/CORR.0000000000002465 36374576 PMC10194687

[2] Veronese N Maggi S . Epidemiology and social costs of hip fracture. Injury. 2018;49(8):1458-1460. doi:10.1016/j.injury.2018.04.015 29699731

[3] Meinberg EG Agel J Roberts CS Karam MD Kellam JF . Fracture and dislocation classification compendium—2018. J Orthop Trauma. 2018;32:S1-S170. doi:10.1097/BOT.0000000000001063 29256945

[4] Lewis SR Macey R Gill JR Parker MJ Griffin XL . Cephalomedullary nails versus extramedullary implants for extracapsular hip fractures in older adults. Cochrane Database Syst Rev. 2022;1(1):CD000093. doi:10.1002/14651858.CD000093.pub6 35080771 PMC8791231

[5] Abdulkareem I . A review of tip apex distance in dynamic hip screw fixation of osteoporotic hip fractures. Niger Med J. 2012;53(4):184-191. doi:10.4103/0300-1652.107550 23661875 PMC3640236

[6] Xiong R Mai QG Yang CL Ye SX Zhang X Fan SC . Intramedullary nailing for femoral shaft fractures in adults. Cochrane Database Syst Rev. 2018;2018:CD010524. doi:10.1002/14651858.CD010524.pub2

[7] Curtis MJ Jinnah RH Wilson V Cunningham BW . Proximal femoral fractures: a biomechanical study to compare intramedullary and extramedullary fixation. Injury. 1994;25(2):99-104. doi:10.1016/0020-1383(94)90111-2 8138307

[8] Jones HW Johnston P Parker M . Are short femoral nails superior to the sliding hip screw? A meta-analysis of 24 studies involving 3,279 fractures. Int Orthop. 2006;30(2):69-78. doi:10.1007/s00264-005-0028-0 16496147 PMC2532072

[9] Palm H Jacobsen S Sonne-Holm S Gebuhr P Hip Fracture Study Group . Integrity of the lateral femoral wall in intertrochanteric hip fractures: an important predictor of a reoperation. J Bone Jt Surg. 2007;89(3):470-475. doi:10.2106/JBJS.F.00679 17332094

[10] Lone DBA Mushtaq DS Shabir DS . Comparative study on evaluation of results of DHS versus PFN in management of intertrochanteric fractures femur. Int J Orthop Sci. 2024;10(1):80-84. doi:10.22271/ortho.2024.v10.i1b.3497

[11] Shen L Zhang Y Shen Y Cui Z . Antirotation proximal femoral nail versus dynamic hip screw for intertrochanteric fractures: a meta-analysis of randomized controlled studies. Orthop Traumatol Surg Res. 2013;99(4):377-383. doi:10.1016/j.otsr.2012.12.019 23707739

[12] Xu H Liu Y Sezgin EA , et al. Comparative effectiveness research on proximal femoral nail versus dynamic hip screw in patients with trochanteric fractures: a systematic review and meta-analysis of randomized trials. J Orthop Surg. 2022;17(1):292. doi:10.1186/s13018-022-03189-z PMC9164432 35658909

[13] Zhang K Zhang S Yang J , et al. Proximal femoral nail vs. dynamic hip screw in treatment of intertrochanteric fractures: a meta-analysis. Med Sci Monit. 2014;20:1628-1633. doi:10.12659/MSM.890962 25213190 PMC4170652

[14] Yu X Wang H Duan X Liu M Xiang Z . Intramedullary versus extramedullary internal fixation for unstable intertrochanteric fracture, a meta-analysis. Acta Orthop Traumatol Turc. 2018;52(4):299-307. doi:10.1016/j.aott.2018.02.009 29602699 PMC6150441

[15] Wessels JO Bjarnesen MP Erichsen JL Palm H Gundtoft PH Viberg B . Sliding hip screw vs intramedullary nail for AO/OTA31A1-A3: a systematic review and meta-analysis. Injury. 2022;53(3):1149-1159. doi:10.1016/j.injury.2021.12.034 35027220

[16] Yoon YC Kim CH Kim YC Song HK . Cephalomedullary nailing versus dynamic hip screw fixation in basicervical femoral neck fracture: a systematic review and meta-analysis. Yonsei Med J. 2022;63(8):744-750. doi:10.3349/ymj.2022.63.8.744 35914756 PMC9344276

[17] Ma Jxiong Kuang Mjie Fan Zrui , et al. Comparison of clinical outcomes with InterTan vs Gamma nail or PFNA in the treatment of intertrochanteric fractures: a meta-analysis. Sci Rep. 2017;7(1):15962. doi:10.1038/s41598-017-16315-3 29162931 PMC5698321

[18] Huang X Leung F Xiang Z , et al. Proximal femoral nail versus dynamic hip screw fixation for trochanteric fractures: a meta-analysis of randomized controlled trials. Sci World J. 2013;2013:1-8. doi:10.1155/2013/805805 PMC3590640 23533361

[19] Audigé L Hanson B Swiontkowski MF . Implant-related complications in the treatment of unstable intertrochanteric fractures: meta-analysis of dynamic screw-plate versus dynamic screw-intramedullary nail devices. Int Orthop. 2003;27(4):197-203. doi:10.1007/s00264-003-0457-6 12734684 PMC3458474

[20] Parker MJ Pryor GA . Gamma versus DHS nailing for extracapsular femoral fractures. Int Orthop. 1996;20(3):163-168. doi:10.1007/s002640050055 8832319

[21] Page MJ McKenzie JE Bossuyt PM , et al. The PRISMA 2020 statement: an updated guideline for reporting systematic reviews. BMJ. 2021;29:n71. doi:10.1136/bmj.n71 PMC8005924 33782057

[22] Higgins J Thomas J Chandler J , et al. Cochrane Handbook for Systematic Reviews of Interventions. Cochrane Collaboration; 2022.

[23] Papp S Backman C Konikoff L , et al. Comparing intramedullary nails versus dynamic hip screws in the treatment of intertrochanteric hip fractures on post-operative rehabilitation outcomes:A systematic review protocol. Geriatr Orthop Surg Rehabil. 2022;13:215145932211441. doi:10.1177/21514593221144180 PMC9726835 36507114

[24] Fracture and dislocation compendium. Orthopaedic trauma association committee for coding and classification. J Orthop Trauma. 1996;10(Suppl 1):1-154. https://pubmed.ncbi.nlm.nih.gov/8814583/. Accessed May 20, 2025. 8814583

[25] Müller ME Nazarian S Koch P . Classification AO des fractures. 1. In: Les Os Longs. Springer-Verlag; 1987.

[26] Müller ME Nazarian S Koch P Schatzker J . The Comprehensive Classification of Fractures of Long Bones. Springer Science & Business Media; 1990.

[27] Marsh JL Slongo TF Agel J , et al. Fracture and dislocation classification compendium - 2007: orthopaedic trauma association classification, database and outcomes committee. J Orthop Trauma. 2007;21(10):S1-S133. 18277234 10.1097/00005131-200711101-00001

[28] McGowan J Sampson M Salzwedel DM Cogo E Foerster V Lefebvre C . PRESS peer review of electronic search strategies: 2015 guideline statement. J Clin Epidemiol. 2016;75:40-46. doi:10.1016/j.jclinepi.2016.01.021 27005575

[29] Sterne JAC Savović J Page MJ , et al. RoB 2: a revised tool for assessing risk of bias in randomised trials. BMJ. 2019;28:l4898. doi:10.1136/bmj.l4898 31462531

[30] Schünemann HJ Cuello C Akl EA , et al. GRADE guidelines: 18. How ROBINS-I and other tools to assess risk of bias in nonrandomized studies should be used to rate the certainty of a body of evidence. J Clin Epidemiol. 2019;111:105-114. doi:10.1016/j.jclinepi.2018.01.012 29432858 PMC6692166

[31] McGuinness LA Higgins JPT . Risk‐of‐bias VISualization (robvis): an R package and Shiny web app for visualizing risk‐of‐bias assessments. Res Synth Methods. 2021;12(1):55-61. doi:10.1002/jrsm.1411 32336025

[32] The Cochrane Collaboration . Review Manager (RevMan) [Computer program]. Version 5.4. 2021. https://training.cochrane.org/online-learning/core-software/revman. Accessed April 29, 2024.

[33] Tufanaru C Munn Z Stephenson M Aromataris E . Fixed or random effects meta-analysis? Common methodological issues in systematic reviews of effectiveness. Int J Evid Based Healthc. 2015;13(3):196-207. doi:10.1097/XEB.0000000000000065 26355603

[34] Deeks JJ Higgins JP Altman DG . In: Thomas J Chandler J Cumpston M Li T Page MJ Welch VA , eds. Chapter 10: Analysing Data and Undertaking Meta-Analyses. Cochrane Collaboration; 2022. doi:10.1002/9781119536604.ch10

[35] Adams CI Robinson CM Court-Brown CM McQueen MM . Prospective randomized controlled trial of an intramedullary nail versus dynamic screw and plate for intertrochanteric fractures of the femur. J Orthop Trauma. 2001;15(6):394-400. 11514765 10.1097/00005131-200108000-00003

[36] Adeel K Nadeem RD Akhtar M Sah RK Mohy-Ud-Din I . Comparison of proximal femoral nail (PFN) and dynamic hip screw (DHS) for the treatment of AO A2 and A3 fractures of femur. J Pak Med Assoc. 2020;70(5):815-819. doi:10.5455/JPMA.295426 32400733

[37] Adulkasem N Phinyo P Khorana J Pruksakorn D Apivatthakakul T . Prognostic factors of 1-year postoperative functional outcomes of older patients with intertrochanteric fractures in Thailand: a retrospective cohort study. Int J Environ Res Public Health. 2021;18(13):6896. doi:10.3390/ijerph18136896 34199045 PMC8297186

[38] Ahmad T Muhammad ZA Habib A . Injury specific trauma registry: outcomes of a prospective cohort with proximal femur fractures. Ann Med Surg. 2019;45(101616869):54-58. doi:10.1016/j.amsu.2019.07.015 PMC6642254 31360461

[39] Ahrengart L Tornkvist H Fornander P , et al. A randomized study of the compression hip screw and gamma nail in 426 fractures. Clin Orthop. 2002;401:209-222. 10.1097/00003086-200208000-00024 12151898

[40] Aktselis I Kokoroghiannis C Fragkomichalos E , et al. Prospective randomised controlled trial of an intramedullary nail versus a sliding hip screw for intertrochanteric fractures of the femur. Int Orthop. 2014;38(1):155-161. doi:10.1007/s00264-013-2196-7 24318319 PMC3890147

[41] Alessio-Mazzola M Traverso G Coccarello F Sanguineti F Formica M . Dynamic hip screw versus intramedullary nailing for the treatment of A1 intertrochanteric fractures: a retrospective, comparative study and cost analysis. Jt Dis Relat Surg. 2022;33(2):314-322. doi:10.52312/jdrs.2022.646 35852189 PMC9361108

[42] Amini MH Feldman JJ Weinlein JC 4th . High complication rate in young patients with high-energy intertrochanteric femoral fractures. Orthopedics. 2017;40(2):e293-e299. doi:10.3928/01477447-20161128-04 27925642

[43] Andalib A Etemadifar M Yavari P . Clinical outcomes of intramedullary and extramedullary fixation in unstable intertrochanteric fractures: a randomized clinical trial. Arch Bone Jt Surg. 2020;8(2):190-197. doi:10.22038/abjs.2019.34942.1919 32490050 PMC7191988

[44] Anjum RS Iqbal M Asif S Chaudhary SA Khan MN . Comparison of functional outcome of proximal femoral nail (PFN) and Dynamic hip screw (DHS) in intertrochanteric fractures of femur under spinal anesthesia. Pakistan Medical Forum; 2020:Vol. 31(10), 114-118.

[45] Anshul G Mansi G Sheela J Rajesh M . To evaluate the results of dynamic hip screw (DHS) & proximal femoral nail (PFN) in intertrochanteric fractures of proximal femur with special reference to surgical site infection international journal of toxicological and pharmacological research introduction. Int J Toxicol Pharmacol Res. 2022;12(5):1-9.

[46] Aros B Tosteson ANA Gottlieb DJ Koval KJ . Is a sliding hip screw or im nail the preferred implant for intertrochanteric fracture fixation? Clin Orthop. 2008;466(11):2827-2832. doi:10.1007/s11999-008-0285-5 18465180 PMC2565060

[47] Bajpai H Singh SK Gupta P . A study on comparison of results of proximal femoral nail and DHS in unstable proximal femoral fractures. J Evol Med Dent Sci. 2019;8(7):441-446. doi:10.14260/jemds/2019/97

[48] Balusamy R Kolundan K Anbu S . A comparative study of functional and radiological outcome of pertrochanteric fracture in elderly patients treated with dynamic HIP screw and proximal femoral nail. J Evol Med Dent Sci. 2017;6(5):387-394. doi:10.14260/Jemds/2017/87

[49] Barton TM Gleeson R Topliss C Greenwood R Harries WJ Chesser TJ . A comparison of the long gamma nail with the sliding hip screw for the treatment of AO/OTA 31-A2 fractures of the proximal part of the femur. J Bone Jt Surg-Am. 2010;92(4):792-798. doi:10.2106/JBJS.I.00508 20360500

[50] Biakto KT Paturusi IA Azis HS Putra LT Kurniawan J . A comparison of walking ability between the dynamic hip screw and cephalomedullary nailing fixations in intertrochanteric femur fracture. Bali Med J. 2022;11(1):368-372. doi:10.15562/bmj.v11i1.3207

[51] Bienkowski P Reindl R Berry GK Iakoub E Harvey EJ . A new intramedullary nail device for the treatment of intertrochanteric hip fractures: perioperative experience. J Trauma. 2006;61(6):1458-1462. 17159691 10.1097/01.ta.0000200937.12453.fb

[52] Bridle S Patel A Bircher M Calvert P . Fixation of intertrochanteric fractures of the femur. A randomised prospective comparison of the gamma nail and the dynamic hip screw. J Bone Joint Surg Br. 1991;73-B(2):330-334. doi:10.1302/0301-620X.73B2.2005167 2005167

[53] Butt MS Krikler SJ Nafie S Ali MS . Comparison of dynamic hip screw and gamma nail: a prospective, randomized, controlled trial. Injury. 1995;26(9):615-618. 8550169 10.1016/0020-1383(95)00126-t

[54] Cai L Wang T Di L Hu W Wang J . Comparison of intramedullary and extramedullary fixation of stable intertrochanteric fractures in the elderly: a prospective randomised controlled trial exploring hidden perioperative blood loss. BMC Musculoskelet Disord. 2016;17(1):475. 27846888 10.1186/s12891-016-1333-z PMC5109735

[55] Canbeyli ID Cirpar M Oktas B Coban M . Analysis of factors among 30-day and 1-year mortality rates in patients with borderline stable-unstable intertrochanteric hip fracture. Acta Orthop Traumatol Turc. 2021;55(1):16-21. doi:10.5152/j.aott.2021.20071 33650505 PMC7932736

[56] Carulli C Piacentini F Paoli T Civinini R Innocenti M . A comparison of two fixation methods for femoral trochanteric fractures: a new generation intramedullary system vs sliding hip screw. Clin Cases Miner Bone Metab. 2017;14(1):40-47. doi:10.11138/ccmbm/2017.14.1.040 28740524 PMC5505713

[57] Chander SG Kanagasarathy K Chander V Reddy MN . Treatment of unstable intertrochanteric fracture of femur with dynamic HIP screw versus proximal femoral nail: a comparative study. J Evol Med Dent Sci-JEMDS. 2015;4(59):10284-10293. doi:10.14260/jemds/2015/1482

[58] Chechik O Amar E Khashan M , et al. Favorable radiographic outcomes using the expandable proximal femoral nail in the treatment of hip fractures - a randomized controlled trial. J Orthop. 2014;11(2):103-109. doi:10.1016/j.jor.2014.04.004 25104895 PMC4118564

[59] Chen K Chen S Medicine JYJof C 2018 U . Efficacy of proximal femoral nail anti-rotation and dynamic hip screw internal fixation in the treatment of hip fracture in the elderly patients. E-Centuryus. 2018;11(4):4188-4192.

[60] Cho HM Lee K . Clinical and functional outcomes of treatment for type A1 intertrochanteric femoral fracture in elderly patients: comparison of dynamic hip screw and proximal femoral nail antirotation. Hip Pelvis. 2016;28(4):232-242. doi:10.5371/hp.2016.28.4.232 28097113 PMC5240315

[61] Chua ITH Rajamoney GN Kwek EBK . Cephalomedullary nail versus sliding hip screw for unstable intertrochanteric fractures in elderly patients. J Orthop Surg. 2013;21(3):308-312. 10.1177/230949901302100309 24366790

[62] Cordero-Ampuero J Peix C Marcos S Cordero G-GE . Influence of surgical quality (according to postoperative radiography) on mortality, complications and recovery of walking ability in 1425 hip fracture patients. Injury. 2021;52(Suppl 4):S32-S36. doi:10.1016/j.injury.2021.02.037 33642085

[63] Davis TR Sher JL Checketts RG Porter BB . Intertrochanteric fractures of the femur: a prospective study comparing the use of the Kuntscher-Y nail and a sliding hip screw. Injury. 1988;19(6):421-426. 3267650 10.1016/0020-1383(88)90138-6

[64] De Groof E Pimontel P Boghemans J . Internal fixation of fractures of the proximal femur dynamic hip screw versus nail-plate fixation. Acta Orthop Belg. 1988;54(4):458-464. 3250201

[65] Duan W Wu Y Liu G Chen J . Comparison of the curative effects of PFNA and DHS fixation in treating intertrochanteric fractures in elderly patients; 2017.

[66] Dujardin FH Benez C Polle G Alain J Biga N Thomine JM . Prospective randomized comparison between a dynamic hip screw and a mini-invasive static nail in fractures of the trochanteric area: preliminary results. J Orthop Trauma. 2001;15(6):401-406. 11514766 10.1097/00005131-200108000-00004

[67] Dunne M Kursumovic K Fisher R Parker M . Comparison of outcomes after different methods of fixation for extracapsular hip fractures: an observational study. Injury. 2021;52(10):3031-3035. doi:10.1016/j.injury.2021.02.050 33642086

[68] Duymus TM Aydogmus S Ulusoy I , et al. Comparison of intra- and extramedullary implants in treatment of unstable intertrochanteric fractures. J Clin Orthop Trauma. 2019;10(2):290-295. doi:10.1016/j.jcot.2018.04.003 30828196 PMC6383078

[69] Elis J Chechik O Maman E Steinberg EL . Expandable proximal femoral nails versus 95degree dynamic condylar screw-plates for the treatment of reverse oblique intertrochanteric fractures. Injury. 2012;43(8):1313-1317. doi:10.1016/j.injury.2012.05.004 22613452

[70] Foss NB Kristensen MT Palm H Kehlet H . Postoperative pain after hip fracture is procedure specific. Br J Anaesth. 2009;102(1):111-116. doi:10.1093/bja/aen345 19059921

[71] Foulongne E Gilleron M Roussignol X Lenoble E Dujardin F . Mini-invasive nail versus DHS to fix pertrochanteric fractures: a case-control study. Orthop Traumatol Surg Res. 2009;95(8):592-598. doi:10.1016/j.otsr.2009.08.007 19945367

[72] Fritz T Hiersemann K Krieglstein C Friedl W . Prospective randomized comparison of gliding nail and gamma nail in the therapy of trochanteric fractures. Arch Orthop TRAUMA Surg. 1999;119(1-2):1-6. doi:10.1007/s004020050345 10076936

[73] Fu CW Chen JY Liu YC Liao KW Lu YC . Dynamic hip screw with trochanter-stabilizing plate compared with proximal femoral nail antirotation as a treatment for unstable AO/OTA 31-A2 and 31-A3 intertrochanteric fractures. BioMed Res Int. 2020;2020(101600173):1896935. doi:10.1155/2020/1896935 32923477 PMC7453265

[74] Garg A Kamboj P Sharma PK Yadav U Siwach RC Kadyan V . Evaluation of functional outcome and comparison of three different surgical modalities for management of intertrochanteric fractures in elderly population. Int J Burns Trauma. 2022;12(1):13-22. 35309106 PMC8918764

[75] Giraud B Dehoux E Jovenin N , et al. Comparaison vis-plaque dynamique et ostéosynthèse intra-médullaire antérograde dans les fractures pertrochantériennes. Rev Chir Orthop Reparatrice Appar Mot. 2005;91(8):732-736. doi:10.1016/S0035-1040(05)84484-8 16552995

[76] Grønhaug KML Dybvik E Matre K Ostman B Gjertsen JE . Intramedullary nail versus sliding hip screw for stable and unstable trochanteric and subtrochanteric fractures: 17,341 patients from the Norwegian Hip Fracture Register. Bone Jt J. 2022;104 B(2):274-282. doi:10.1302/0301-620X.104B2.BJJ-2021-1078.R1 35094569

[77] Guerra MTE Pasqualin S Souza MP Lenz R . Functional recovery of elderly patients with surgically-treated intertrochanteric fractures: preliminary results of a randomised trial comparing the dynamic hip screw and proximal femoral nail techniques. Injury. 2014;45:S26-S31. doi:10.1016/S0020-1383(14)70017-8 25528621

[78] Guo QC Shen Y Zong ZW , et al. Percutaneous compression plate versus proximal femoral nail anti-rotation in treating elderly patients with intertrochanteric fractures: a prospective randomized study. J Orthop Sci. 2013;18(6):977-986. doi:10.1007/s00776-013-0468-0 24085380 PMC3838589

[79] Guo Y Yang HP Dou QJ He XB Yang XF . Efficacy of femoral nail anti-rotation of helical blade in unstable intertrochanteric fracture. Eur Rev Med Pharmacol Sci. 2017;21(3 Suppl):6-11. 28745799

[80] Han C Song X Huang Y Yang G . Clinical efficacy and safety of PFNA and DHS in the treatment of unstable intertrochanteric fractures in elderly patients. Acta Medica Mediterr. 2021;37(1):715-722. doi:10.19193/0393-6384_2021_1_110

[81] Hardy DC Descamps PY Krallis P , et al. Use of an intramedullary hip-screw compared with a compression hip-screw with a plate for intertrochanteric femoral fractures. A prospective, randomized study of one hundred patients. J Bone Joint Surg Am. 1998;80(5):618-630. 9611022 10.2106/00004623-199805000-00002

[82] Harrington P Nihal A Singhania AK Howell FR . Intramedullary hip screw versus sliding hip screw for unstable intertrochanteric femoral fractures in the elderly. Injury. 2002;33(1):23-28. 11879828 10.1016/s0020-1383(01)00106-1

[83] Henzman C Ong K Lau E Seligson D Roberts CS Malkani AL . Complication risk after treatment of intertrochanteric hip fractures in the medicare population. Orthopedics. 2015;38(9):e799-805. doi:10.3928/01477447-20150902-58 26375538

[84] Hoffman CW Lynskey TG . Intertrochanteric fractures of the femur: a randomized prospective comparison of the gamma nail and the ambi hip screw. Aust N Z J Surg. 1996;66(3):151-155. 8639131 10.1111/j.1445-2197.1996.tb01144.x

[85] Huang SG Chen B Zhang Y , et al. Comparison of the clinical effectiveness of PFNA, PFLCP, and DHS in treatment of unstable intertrochanteric femoral fracture. Am J Ther. 2015;24(6):e659-e666. doi:10.1097/MJT.0000000000000346 26488362

[86] Jain P Jain SK . Comparison of the efficacy of hip screw and nailing in intertrochanteric fractures of femur at tertiary care level center: a prospective study. Int J Sci Study. 2015;3(6):24-27. doi:10.17354/ijss/2015/385

[87] Jia L Zhang K Wang ZG Wang L Yang SY Zheng YP . Proximal femoral nail antirotation internal fixation in treating intertrochanteric femoral fractures of elderly subjects. J Biol Regul Homeost Agents. 2017;31(2):329-334. 28685532

[88] Jonnes C Sm S Najimudeen S . Type II intertrochanteric fractures: proximal femoral nailing (PFN) versus dynamic hip screw (DHS). Arch Bone Jt Surg. 2016;4(1):23-28. 26894214 PMC4733231

[89] Khan M Siraj M Ali A . Dynamic hip screw in comparison with proximal femoral nail technique in intertrochanteric femur fracture patients. Pak J Med Health Sci. 2021;15(11):2966-2968. doi:10.53350/pjmhs2115112966

[90] Koduru SK Potluri S Rao CH Raghav TS . Comparative study of proximal femoral nailing and dynamic HIP screw for intertrochanteric fractures in adults. J Evol Med Dent Sci-JEMDS. 2016;5(35):2018-2021. doi:10.14260/jemds/2016/474

[91] Kumar R Singh RN Singh BN . Comparative prospective study of proximal femoral nail and dynamic hip screw in treatment of intertrochanteric fracture femur. J Clin Orthop Trauma. 2012;3(1):28-36. doi:10.1016/j.jcot.2011.12.001 25983453 PMC3876488

[92] Kyavater BS Gupta S . Comparative study between dynamic HIP screw vs proximal femoral nailing in unstable inter-trochanteric fractures of the femur in adults. J Evol Med Dent Sci. 2015;4(50):8690-8693. doi:10.14260/jemds/2015/1257

[93] Leung KS So WS Shen WY Hui PW . Gamma nails and dynamic hip screws for peritrochanteric fractures. A randomised prospective study in elderly patients. J Bone Joint Surg Br. 1992;74(3):345-351. 1587874 10.1302/0301-620X.74B3.1587874

[94] Li H Wang Q Dai GG Peng H . PFNA vs. DHS helical blade for elderly patients with osteoporotic femoral intertrochanteric fractures. Eur Rev Med Pharmacol Sci. 2021;22(1):1-7. doi:10.26355/eurrev_201807_15346 30004570

[95] Little NJ Verma V Fernando C Elliott DS Khaleel A . A prospective trial comparing the Holland nail with the dynamic hip screw in the treatment of intertrochanteric fractures of the hip. J Bone Joint Surg Br. 2008;90(8):1073-1078. doi:10.1302/0301-620X.90B8.20825 18669966

[96] Matre K Havelin LI Gjertsen JE Espehaug B Fevang JM . Intramedullary nails result in more reoperations than sliding hip screws in two-part intertrochanteric fractures. Clin Orthop. 2013;471(4):1379-1386. doi:10.1007/s11999-012-2728-2 23224796 PMC3586046

[97] Matre K Havelin LI Gjertsen JE Vinje T Espehaug B Fevang JM . Sliding hip screw versus IM nail in reverse oblique trochanteric and subtrochanteric fractures. A study of 2716 patients in the Norwegian hip fracture register. Injury. 2013;44(6):735-742. doi:10.1016/j.injury.2012.12.010 23305689

[98] Matre K Vinje T Havelin LI , et al. Trigen intertan intramedullary nail versus sliding hip screw. J Bone Jt Surg. 2013;95(3):200-208. doi:10.2106/JBJS.K.01497 23389782

[99] McLaren CA Buckley JR Rowley DI . Intertrochanteric fractures of the femur: a randomized prospective trial comparing the Pugh nail with the dynamic hip screw. Injury. 1991;22(3):193-196. 2071200 10.1016/0020-1383(91)90039-h

[100] Mohan H Kumar P . Surgical treatment of type 31-A1 two-part intertrochanteric femur fractures: is proximal femoral nail superior to dynamic hip screw fixation? Cureus. 2019;11(2):e4110. doi:10.7759/cureus.4110 31058004 PMC6476610

[101] O’Brien PJ Meek RN Blachut PA Broekhuyse HM Sabharwal S . Fixation of intertrochanteric hip fractures: gamma nail versus dynamic hip screw. A randomized, prospective study. Can J Surg. 1995;38(6):516-520. 7497366

[102] Ong JCY Gill JR Parker MJ . Mobility after intertrochanteric hip fracture fixation with either a sliding hip screw or a cephalomedullary nail: sub group analysis of a randomised trial of 1000 patients. Injury. 2019;50(10):1709-1714. doi:10.1016/j.injury.2019.06.015 31256911

[103] Pajarinen J Lindahl J Michelsson O Savolainen V Hirvensalo E . Pertrochanteric femoral fractures treated with a dynamic hip screw or a proximal femoral nail. A randomised study comparing post-operative rehabilitation. J Bone Joint Surg Br. 2005;87(1):76-81. 15686241

[104] Parker MJ Bowers TR Pryor GA . Sliding hip screw versus the Targon PF nail in the treatment of trochanteric fractures of the hip: a randomised trial of 600 fractures. J Bone Joint Surg Br. 2012;94(3):391-397. doi:10.1302/0301-620X.94B3.28406 22371549

[105] Parker MJ . Sliding hip screw versus intramedullary nail for trochanteric hip fractures; a randomised trial of 1000 patients with presentation of results related to fracture stability. Injury. 2017;48(12):2762-2767. doi:10.1016/j.injury.2017.10.029 29102044

[106] Patel VA Sheth CB Bhundiya S Patel SR Patel AV Pavani DM . To compare and assess dynamic hip screw and proximal femur nail in intertrochenteric femur fracture. J Res Med Dent Sci. 2021;9(7):219-224.

[107] Pehlivanoglu T Bayram S Demirel M , et al. Proximal femoral nailing versus dynamic HIP screw in management of stable intertrochanteric femur fractures: a comparison of clinical and radiological outcomes. J Istanb Fac Med-Istanb TIP Fak Derg. 2021;84(4):514-520. doi:10.26650/IUITFD.2021.964078

[108] Prakash AK Shanthappa AH Venkataraman S Kamath A . A comparative study of functional outcome following dynamic hip screw and proximal femoral nailing for intertrochanteric fractures of the femur. Cureus. 2022;14(4):e23803. doi:10.7759/cureus.23803 35518518 PMC9066962

[109] Pundkar AG Modi NS Baitule RW Pundkar GN . Evaluation of dynamic hip screw plate v/s proximal femoral nail for unstable inter-trochanteric fracture femur. J Res Med Dent Sci. 2016;4(3):283-287. doi:10.5455/jrmds.20164323

[110] Radcliff TA Regan E Ripley DCC Hutt E . Increased use of intramedullary nails for intertrochanteric proximal femoral fractures in veterans affairs hospitals A comparative effectiveness study. J BONE Jt Surg-Am. 2012;94A(9):833-840. doi:10.2106/JBJS.I.01403 22552673

[111] Rao DV Kumar CS Sangepu A . Surgical management of intertrochanteric fractures: a study using dynamic HIP screw and proximal femoral nail. J Evol Med Dent Sci-JEMDS. 2015;4(66):11440-11445. doi:10.14260/jemds/2015/1651

[112] Reddy PS Ponugoti N Jampana VS . A comparative study of reconstruction nailing versus dynamic HIP screw device in the surgical management of intertrochanteric fractures. J Evol Med Dent Sci-JEMDS. 2016;5(99):7224-7230. doi:10.14260/jemds/2016/1635

[113] Reindl R Harvey EJ Berry GK Rahme E Cots COTS . Intramedullary versus extramedullary fixation for unstable intertrochanteric fractures: a prospective randomized controlled trial. J Bone Joint Surg Am. 2015;97(23):1905-1912. doi:10.2106/JBJS.N.01007 26631990

[114] Rogmark C Spetz CL Garellick G . More intramedullary nails and arthroplasties for treatment of hip fractures in Sweden. Acta Orthop. 2010;81(5):588-592. doi:10.3109/17453674.2010.506631 20860442 PMC3214748

[115] Sanders D Bryant D Tieszer C , et al. A multicenter randomized control trial comparing a novel intramedullary device (InterTAN) versus conventional treatment (sliding hip screw) of geriatric hip fractures. J Orthop Trauma. 2017;31(1):1-8. doi:10.1097/BOT.0000000000000713 27763958

[116] Saudan M Lübbeke A Sadowski C Riand N Stern R Hoffmeyer P . Petrochanteric fractures: is there an advantage to an intramedullary nail? A randomized, prospective study of 206 patients comparing the dynamic hip screw and proximal femoral nail. J Orthop Trauma. 2002;16(6):386-393. doi:10.1097/00005131-200207000-00004 12142826

[117] Schemitsch EH Nowak LL Schulz AP , et al. Intramedullary nailing vs sliding hip screw in trochanteric fracture management: the INSITE randomized clinical trial. JAMA Netw Open. 2023;6(6):e2317164. doi:10.1001/jamanetworkopen.2023.17164 37278998 PMC10245197

[118] Sevinc HF Cirpar M Canbeyli ID Daglar B Oktas B Durusoy S . Comparison of functional outcomes in patients fixed with dynamic hip screw and proximal femur nail-anti-rotation in A1 and A2 type intertrochanteric femur fractures. Ulus Travma Ve Acil Cerrahi Derg Turk J Trauma Emerg Surg TJTES. 2020;26(5):811-817. doi:10.14744/tjtes.2020.39888 32946090

[119] Sharma A Sethi A Sharma S . Treatment of stable intertrochanteric fractures of the femur with proximal femoral nail versus dynamic hip screw: a comparative study. Rev Bras Ortop. 2018;53(4):477-481. doi:10.1016/j.rboe.2017.07.008 30027082 PMC6052185

[120] Shishodia AS Dakour VK Bhatia R . A prospective study comparing the outcome of dynamic hip screw and proximal femoral nail in the treatment of intertrochanteric fractures of femur. Indian J Public Health Res Dev. 2017;8(2):100. doi:10.5958/0976-5506.2017.00091.2

[121] Shivanna UM Rudrappa GH . A comparative study of functional outcome between dynamic HIP screw and proximal femoral nail in surgical management of per-trochanteric fractures. J Evol Med Dent Sci. 2015;4(43):7489-7498. doi:10.14260/jemds/2015/1087

[122] Singh D Singh A Singh G Singh M Sandhu A Sandhu KS . Comparative study of the management of intertrochanteric fracture femur with proximal femoral nail vs. the dynamic hipscrew with derotation screw in elderly population. Cureus. 2021;13:e19431. doi:10.7759/cureus.19431 34926021 PMC8654079

[123] Singh NK Sharma V Trikha V , et al. Is PFNA-II a better implant for stable intertrochanteric fractures in elderly population ? A prospective randomized study. J Clin Orthop Trauma. 2019;10(Suppl 1):S71-S76. doi:10.1016/j.jcot.2019.02.004 31700206 PMC6823828

[124] Suh YS Nho JH Kim SM Hong S Choi HS Park JS . Clinical and radiologic outcomes among bipolar hemiarthroplasty, compression hip screw and proximal femur nail antirotation in treating comminuted intertrochanteric fractures. Hip Pelvis. 2015;27(1):30-35. doi:10.5371/hp.2015.27.1.30 27536599 PMC4972617

[125] Taqi M Tasneem M Akhtar M , et al. Treatment and outcomes of proximal femoral locking plate versus proximal femoral nail in unstable pert-trochanteric fractures (Boyd and Griffin Type III & IV). Pakistan Journal of Medical and Health Sciences; 2021:Vol. 15(1), 133-136.

[126] Tucker A Donnelly KJ Rowan C McDonald S Foster AP . Is the best plate a nail? A review of 3230 unstable intertrochanteric fractures of the proximal femur. J Orthop Trauma. 2018;32(2):53-60. doi:10.1097/BOT.0000000000001038 29040233

[127] Utrilla AL Reig JS Munoz FM Tufanisco CB . Trochanteric gamma nail and compression hip screw for trochanteric fractures: a randomized, prospective, comparative study in 210 elderly patients with a new design of the gamma nail. J Orthop Trauma. 2005;19(4):229-233. 15795570 10.1097/01.bot.0000151819.95075.ad

[128] Verettas DAJ Ifantidis P Chatzipapas CN , et al. Systematic effects of surgical treatment of hip fractures: gliding screw-plating vs intramedullary nailing. Injury. 2010;41(3):279-284. doi:10.1016/j.injury.2009.09.012 20176167

[129] Wang B Liu Q Liu Y Jiang R . Comparison of proximal femoral nail antirotation and dynamic hip screw internal fixation on serum markers in elderly patients with intertrochanteric fractures. J Coll Physicians Surg Pak. 2019;29(7):644-648. doi:10.29271/jcpsp.2019.07.644 31253216

[130] Warren JA Sundaram K Hampton R , et al. Cephalomedullary nailing versus sliding hip screws for Intertrochanteric and basicervical hip fractures: a propensity-matched study of short-term outcomes in over 17,000 patients. Eur J Orthop Surg Traumatol. 2020;30(2):243-250. doi:10.1007/s00590-019-02543-y 31486944

[131] Whale CS Hulet DA Beebe MJ , et al. Cephalomedullary nail versus sliding hip screw for fixation of AO 31 A1/2 intertrochanteric femoral fracture: a 12-year comparison of failure, complications, and mortality. Curr Orthop Pract. 2016;27(6):604-613. doi:10.1097/BCO.0000000000000424 28348717 PMC5364496

[132] Wolf O Mukka S Ekelund J Rogmark C Moller M Hailer NP . Increased mortality after intramedullary nailing of trochanteric fractures: a comparison of sliding hip screws with nails in 19,935 patients. Acta Orthop. 2022;93(101231512):146-150. doi:10.2340/17453674.2021.862 34984474 PMC8815803

[133] Xu Y Geng D Mao H Zhu X Yang H . A comparison of the proximal femoral nail antirotation device and dynamic hip screw in the treatment of unstable pertrochanteric fracture. J Int Med Res. 2010;38(4):1266-1275. doi:10.1177/147323001003800408 20925999

[134] Yamauchi K Fushimi K Shirai G Fukuta M . Comparison of functional recovery in the very early period after surgery between plate and nail fixation for correction of stable femoral intertrochanteric fractures: a controlled clinical trial of 18 patients. Geriatr Orthop Surg Rehabil. 2014;5(2):63-68. doi:10.1177/2151458514527607 25360333 PMC4212367

[135] Yeganeh A Taghavi R Moghtadaei M . Comparing the intramedullary nailing method versus dynamic hip screw in treatment of unstable intertrochanteric fractures. Med Arch. 2016;70(1):53-56. doi:10.5455/medarh.2016.70.53-56 26980933 PMC4779359

[136] Yu W Zhang X Wu R , et al. The visible and hidden blood loss of Asia proximal femoral nail anti-rotation and dynamic hip screw in the treatment of intertrochanteric fractures of elderly high- risk patients: a retrospective comparative study with a minimum 3 years of follow-up. BMC Musculoskelet Disord. 2016;17(1):269. doi:10.1186/s12891-016-1143-3 27401011 PMC4940845

[137] Yu W Zhang X Zhu X , et al. Proximal femoral nails anti-rotation versus dynamic hip screws for treatment of stable intertrochanteric femur fractures: an outcome analyses with a minimum 4 years of follow-up. BMC Musculoskelet Disord. 2016;17(1):222. doi:10.1186/s12891-016-1079-7 27209256 PMC4875726

[138] Zang W Liu PF Han XF . A comparative study of proximal femoral locking compress plate, proximal femoral nail antirotation and dynamic hip screw in intertrochanteric fractures. Eur Rev Med Pharmacol Sci. 2018;22(1 Suppl):119-123. doi:10.26355/eurrev_201807_15373 30004556

[139] Zehir S Zehir R Zehir S Azboy I Haykir N . Proximal femoral nail antirotation against dynamic hip screw for unstable trochanteric fractures; a prospective randomized comparison. Eur J Trauma Emerg Surg. 2015;41(4):393-400. doi:10.1007/s00068-014-0463-y 26037995

[140] Zeng X Zhang N Zeng D , et al. Proximal femoral nail antirotation versus dynamic hip screw fixation for treatment of osteoporotic type 31-A1 intertrochanteric femoral fractures in elderly patients. J Int Med Res. 2017;45(3):1109-1123. doi:10.1177/0300060517703277 28417681 PMC5536426

[141] Ziran BH Heckman DS Olarte CM Chou K Baranick J . Intramedullary hip screw versus standard compression hip screw: early postoperative rehabilitation comparisons. Orthopedics. 2009;32(2):83. 19301807

[142] Zou J Xu Y Yang H . A comparison of proximal femoral nail antirotation and dynamic hip screw devices in trochanteric fractures. J Int Med Res. 2009;37(4):1057-1064. 19761688 10.1177/147323000903700410

[143] Swart E Makhni EC Macaulay W Rosenwasser MP Bozic KJ . Cost-effectiveness analysis of fixation options for intertrochanteric hip fractures. J Bone Jt Surg. 2014;96(19):1612-1620. doi:10.2106/JBJS.M.00603 25274786

[144] O’Connor MI Switzer JA . AAOS clinical practice guideline summary: management of hip fractures in older adults. J Am Acad Orthop Surg. 2022;30(20):e1291-e1296. doi:10.5435/JAAOS-D-22-00125 36200817

[145] Yu F Tang YW Wang J Lin ZC Liu YB . Does intramedullary nail have advantages over dynamic hip screw for the treatment of AO/OTA31A1-A3? A meta-analysis. BMC Musculoskelet Disord. 2023;24(1):588. doi:10.1186/s12891-023-06715-0 37464358 PMC10355055

[146] Raj S Grover S Bola H Pradhan A Fazal MA Patel A . Dynamic hip screws versus cephalocondylic intramedullary nails for unstable extracapsular hip fractures in 2021: a systematic review and meta-analysis of randomised trials. J Orthop. 2023;36:88-98. doi:10.1016/j.jor.2022.12.015 36654796 PMC9841034

[147] Page P Lord R Jawad A , et al. Changing trends in the management of intertrochanteric hip fractures - a single centre experience. Injury. 2016;47(7):1525-1529. doi:10.1016/j.injury.2016.05.002 27222104

[148] Berney M Moore J Walsh M , et al. Is the increased use of intramedullary nailing over DHS for intertrochanteric hip fractures justified? – a review of the Irish hip fracture database 2016 –2020. Surgeon. 2024;22:31-36. doi:10.1016/j.surge.2023.09.002 37793947

[149] Zhu Q Xu X Yang X , et al. Intramedullary nails versus sliding hip screws for AO/OTA 31-A2 trochanteric fractures in adults: a meta-analysis. Int J Surg. 2017;43:67-74. doi:10.1016/j.ijsu.2017.05.042 28549994

[150] The American Academy of Orthopaedic Surgeons Board of Directors . American academy of orthopaedic surgeons management of hip fractures in older adults evidence based clinical practice guideline. 2021. https://www.aaos.org/globalassets/quality-and-practice-resources/hip-fractures-in-the-elderly/hipfxcpg.pdf 10.5435/JAAOS-D-22-00273 36200818

[151] Health Quality Ontario . Hip fracture: care for people with fragility fractures. 2017. https://www.hqontario.ca/portals/0/documents/evidence/quality-standards/qs-hip-fracture-clinical-guide-en.pdf

