# Supplementary material for: Comparing Intramedullary Nails versus Dynamic Hip Screws in the Treatment of Intertrochanteric Hip Fractures on Post-operative Rehabilitation Outcomes – A Systematic Review and Meta-Analysis
Source: Geriatr Orthop Surg Rehabil. 2025 Jun 17;16:21514593251350490. doi: 10.1177/21514593251350490 (PMC12174732; doi:10.1177/21514593251350490)
Supplement: Supplemental Material - Comparing Intramedullary Nails versus Dynamic Hip Screws in the Treatment of Intertrochanteric Hip Fractures on Post-operative Rehabilitation Outcomes – A Systematic Review and Meta-Analysis [file sj-pdf-1-gos-10.1177_21514593251350490.pdf]

Supplemental file #1 – Search Strategies

Ovid MEDLINE(R) ALL

1 exp Hip Fractures/
2 ((intertrochanteric adj3 fracture*) or IFT).ti,ab,kw,kf.
3 (Trochanteric adj3 fracture*).ti,ab,kw,kf.
4 ((Hip? or femur? or femoral) adj3 fracture*).ti,ab,kw,kf.
5 or/1-4
6 Bone Screws/
7 ((Dynamic adj2 Hip adj2 Screw*) or dhs).ti,ab,kw,kf.
8 ((bone or hip?) adj3 (pin? or screw*)).ti,ab,kw,kf.
9 or/6-8
10 Fracture Fixation, Intramedullary/
11 Bone Nails/
12 ((Intramedullary adj3 nail*) or IM or PFN).ti,ab,kw,kf.
13 ((fixation or nail* or rod?) adj3 (intramedullary or interlock* or inter-lock* or kuntscher?)).ti,ab,kw,kf.
14 (Proximal adj3 (nail* or intramedullary)).ti,ab,kw,kf.
15 or/10-14
16 5 and 9 and 15

Embase Classic+Embase (Ovid)

1 exp Hip Fractures/
2 femur intertrochanteric fracture/ or proximal femur fracture/
3 ((intertrochanteric adj3 fracture*) or IFT).ti,ab,kw,kf.
4 (Trochanteric adj3 fracture*).ti,ab,kw,kf.
5 ((Hip? or femur? or femoral) adj3 fracture*).ti,ab,kw,kf.
6 or/1-5
7 dynamic hip screw/
8 exp Bone Screws/
9 ((Dynamic adj2 Hip adj2 Screw*) or dhs).ti,ab,kw,kf.
10 ((bone or hip?) adj3 (pin? or screw*)).ti,ab,kw,kf.
11 or/7-10
12 intramedullary nailing/
13 exp intramedullary nail/
14 ((Intramedullary adj3 nail*) or IM or PFN).ti,ab,kw,kf.
15 ((fixation or nail* or rod?) adj3 (intramedullary or interlock* or inter-lock* or kuntscher?)).ti,ab,kw,kf.
16 (Proximal adj3 (nail* or intramedullary)).ti,ab,kw,kf.
17 or/12-16
18 6 and 11 and 17

EBM Reviews (Ovid) Including Cochrane Database of Systematic Reviews, ACP Journal Club, Database of Abstracts of Reviews of Effects, Cochrane Clinical Answers, Cochrane Central Register of Controlled Trials, Cochrane Methodology Register, Health Technology Assessment, NHS Economic Evaluation Database)

1 ((intertrochanteric adj3 fracture*) or IFT).ti,ab.
2 (Trochanteric adj3 fracture*).ti,ab.
3 ((Hip? or femur? or femoral) adj3 fracture*).ti,ab.
4 or/1-3
5 ((Dynamic adj2 Hip adj2 Screw*) or dhs).ti,ab.
6 ((bone or hip?) adj3 (pin? or screw*)).ti,ab.
7 or/5-6
8 ((Intramedullary adj3 nail*) or IM or PFN).ti,ab.
9 ((fixation or nail* or rod?) adj3 (intramedullary or interlock* or inter-lock* or kuntscher?)).ti,ab.
10 (Proximal adj3 (nail* or intramedullary)).ti,ab.
11 or/8-10
12 4 and 7 and 11
13 remove duplicates from 12

Web of Science Core Collection (Clarivate)

(IFT or (intertrochanteric or Trochanteric or Hip? or femur? or femoral) NEAR/4 fracture*) (Topic) and ("Dynamic Hip Screw*" or dhs or (bone or hip?) NEAR/4 (pin or pins or screw*)) (Topic) and (IM or PFN or (Intramedullary NEAR/4 nail*) or (fixation or nail* or rod or rods) NEAR/4 (intramedullary or interlock* or inter-lock* or kuntscher) or (Proximal NEAR/4 (nail* or intramedullary))) (Topic)
